# The role of embedded Non-Governmental Organisations and other stakeholders in building resilience to cyclone-related crises in Madagascar: a qualitative study

**DOI:** 10.1186/s44263-026-00300-y

**Published:** 2026-07-09

**Authors:** Mateus Kambale Sahani, Vikas Mohan, Neha S. Singh, Janet Seeley, PArtnership for RESilience (PARES) Research Group, Susannah H. Mayhew

**Affiliations:** 1 Department of Global Health and Development, Faculty of Public Health and Policy, London School of Hygiene & Tropical Medicine, London, UK; 2 Independent Researcher, Health Research, Exeter, UK; 3 Department of Health Services Research and Policy, Faculty of Public Health and Policy, London School of Hygiene & Tropical Medicine, London, UK

**Keywords:** Cyclone, Disaster, Climate change, Community engagement, Health shocks, Environmental hazards, Infectious diseases, Ambanja, Madagascar

## Abstract

**Background:**

Madagascar has been seriously affected by climate change with a particular impact on population health, wellbeing, and livelihoods. The country is prone to cyclones with floods as immediate consequences creating a cascade of crises. This study aims to understand how stakeholders (state and Non-Governmental Organisations) engage with communities to respond to cyclone-related shocks, how communities themselves respond, and what factors facilitate or hinder these partnerships.

**Methods:**

This qualitative study collected data though In-Depth Interviews (IDIs) from community members, Key Informant Interviews (KIIs) from stakeholders, and Focus Group Discussions (FGDs) from community members in Ambanja district of Madagascar. Data collection was guided by a realist-informed approach collecting data specific to Context, Mechanisms, and Outcome (CMO) of community engagement and involvement (CEI) for the response to cyclone-related crises. Data were presented as narrative summaries before going thorough framework analysis.

**Results:**

This study identifies three pathways enabling community resilience. First, timely cyclone early-warning communication from trusted stakeholders from local actors and supporting Non-Governmental Organisations (NGOs) facilitates community involvement and saves lives during cyclone events. Second, embedded NGOs who are committed to working with communities play a pivotal role in building trust and delivering jointly long-term interventions essential for sustained resilience. Third, existing community social capital—including local knowledge, labour and solidarity networks—represents vital yet underutilised resources for effective intervention design. However, two fundamental challenges systematically undermine resilience-building efforts. Most significantly, the predominance of short-term partnerships means that only a few NGOs engage in long-term interventions, leaving substantial community needs unmet and limiting resilience to survival rather than at levels to thrive. Additionally, ignoring local knowledge and social norms leads to waste of resource through non-sustainable interventions, exemplified by water facilities that were rapidly destroyed because stakeholders failed to incorporate community insights about flood-resistant design.

**Conclusions:**

Whilst communities possess considerable adaptive capacity, and strong social solidarity, greater resilience requires sustained institutional commitment to longer-term engagement and support to address community needs. This needs a systematic integration of existing community knowledge and networks, rather than continued reliance on short-term, externally designed interventions that neither build upon local strengths nor address underlying vulnerabilities.

**Supplementary Information:**

The online version contains supplementary material available at 10.1186/s44263-026-00300-y.

## Background

Madagascar is seriously affected by climate change with a particular impact on the population’s health, wellbeing, and food production leading to food insecurity and exacerbating the risk of hunger and malnutrition in the country [1–3]. Rice production has dropped by 75% exacerbating the malnutrition rate in the country [4], bringing the rate of child malnutrition to one malnourished child out of two [3]. Researchers have shown that the increase in temperature and multiple floods resulting from tropical cyclones have also been the source of multiple cholera outbreaks [4].

Climate change hazards, specifically cyclones, negatively impact Madagascar in diverse sectors. First, health is affected by environmental degradation that provides favourable conditions to vectors that carry micro-organisms responsible of zoonotic diseases [1, 5, 6] and other infectious diseases [7, 8]. Second, water sources are impacted exposing people to not only the risk of water shortages for agricultural activities and domestic use, but also the risk of the consumption of contaminated water after heavy rain exacerbating the exposure to infectious diseases [5, 9, 10]. Third, the agriculture sector is affected with immediate impacts on food production leading to food insecurity and exacerbating difficult living conditions [2, 6, 7, 11].

Tropical cyclones often lead to floods which are among the major hazards of societal concern [12, 13], yet this hazard is not getting enough attention from the scientific and professional communities to support affected people [12, 13]. One major impact is cyclone-related flooding which contaminates water sources [5, 14, 15] and affects livelihoods as most people depend on agriculture for their subsistence [6]. This has obliged people to adopt different approaches, including behaviour changes, towards water treatment methods [16]. Despite advice to treat water before use, the rate of people doing this is still low (up to 65%) [16] exposing people to the risk of infectious disease outbreaks. Studies have shown that episodes of heavy rainfall and population movement to urban areas of Madagascar have led to floods increasing water source contamination with bacteria of faecal origin [5, 15, 17]. Climate change is expected to have more impact on water sources which are unevenly distributed across Madagascar increasing the burden on the health sector and agriculture [18]. Moreover, severe flooding has wider consequences, creating social isolation as families and villages are cut-off from others, and disrupts healthcare and humanitarian programmes, increasing the strain on critical infrastructure and diseases [19–21].

The flooding and landslides that many cyclones leave behind intensified pre-existing stressors on communities by decreasing their means of subsistence (agricultural and fisheries activities were often negatively impacted) which increases poverty and leads to loss of hope in affected communities, which can struggle to recover for many months following a cyclone [8]. Sometimes, a new climate adverse event occurred while the effects of the previous one was not yet fully recovered from and this situation pushed people to leave their usual place of living to settle in new places (mostly uphill) looking for a new life [8, 22]. The recurrent crop failure in addition to floods and landslides are factors that lock some regions in poverty and maintain people in long-lasting stress, hunger and malnutrition, specifically in Southwestern Madagascar [23].

In extreme contexts such as Madagascar, disaster-recovery is very difficult and the country lacks the capacity to adapt to and recover from the negative impacts of climate change and ensure the resilience of local populations [18]. In this paper we define resilience as: the capacity to withstand or to recover quickly from difficult situations [22–24]. In Madagascar, as in many very poor countries, the State is unable, and at times unwilling, to provide support to build resilience to repeated crises. In these settings, communities and other non-state actors particularly Non-Governmental Organisations (NGOs) play a critical role.

The role of community in responding to climate-related crises in Madagascar has been particularly relevant with community members supporting each other in difficult moments even without robust aid relief [25]. Communities can provide powerful social capital to support individuals when no other support is available. Daniel Aldrich’s work has shown that high levels of social capital are more effective in building community resilience and achieving post-disaster recovery than greater economic resources or assistance from external agencies [26, 27]. However, much of his work has been in high- and middle-income settings rather than those of extreme poverty or those facing recurrent disasters like Madagascar which is ranked 13th most vulnerable and 173rd for adaptation on the list of Notre Dame Vulnerability Index [28]. In low-income settings non-state actors play an increasingly important role in the provision of public goods and across Africa, citizens have reported that NGOs, traditional leaders and community leaders are big providers of public goods including healthcare and social welfare [29]. It has been argued that this is due to a combination of factors, including weak state capacity, spread of market economies and democratisation which enables freedom of provision beyond state control [29, 30]. The wider consequences of the actions of nonstate actors are mixed and uneven yet often under-researched with little known about whether and how they build social capital or community level crisis-resilience, and there are calls for more empirical studies of the breadth and depth of nonstate provision at community level, its causes and consequences [29, 31].

In Madagascar, community action certainly constitutes a great resource for the country to build resilience to climate change crises [18]. Several studies have shown different aspects of community engagement to be important in building resilience and effective responses to crises. Building and strengthening local capacity to cope with and adapt to the immediate and long-term effects of climate hazards has been found to be very important for communities where the majority of people rely on natural resources for their life and subsistence [32]. One of the effective strategies to build social resilience at the community level has been found to be the involvement of community members in the whole journey of the response including the planning steps and keeping constant contact with the community in time of crisis [33]. Research on Social and Behaviour Change (SBC) communication in Madagascar to assess the communication impact on preparedness of communities before, during, and after cyclone Freddy showed positive results when communication messages were redefined by integrating the community feedback (consulting community members) to increase the level of community engagement [34]. One study in marine-dependent communities, showed the need to assess and integrate local needs from the beginning to ensure effective implementation of the response and ownership of the process [35]. Another study has shown how working jointly with communities as stakeholders, investing in agroforestry for natural resources restoration, is an effective way to mitigate climate impact and achieve community development [36]. Another study showed that the resilience of community members could be built through planning and implementing projects that enhance and strengthen technical capacities of the local community such as increasing their capacity for producing foods, management and maintenance of the marine environment for fisheries conservation and joint planning for the conservation of protected areas [37].

Despite studies showing the clear importance of engaging and involving communities in crisis-response, few studies give a detailed understanding of the roles of the different stakeholders (usually non-state) or how and why different types of community engagement or collaboration are effective. It is not clear from literature how different kinds of social capital, like local community knowledge or local manpower, is or should be harnessed to contribute towards resilience to climate-related crises. Information about community coping mechanisms (another form of social capital) to withstand the repeated cyclone shocks is missing. There is also limited evidence on how local community knowledge sheds light on future project planning for thriving resilience.

A French translation of the abstract is provided in Supplementary material 1.

This research aimed to explore how stakeholders (governments and non-state actors, particularly NGOs) engage with the communities to respond to climate cyclone-related shocks, how communities themselves respond to cyclone crises, and what factors facilitate or hinder these partnerships. Hence, the following research questions are investigated in this study. First, how have communities and local leaders affected by cyclone crises responded to environmental and/or health shocks in Ambanja district in Madagascar? Second, how do government and NGOs/ other stakeholders engage with community members to implement cyclone crisis-responses and what role do the NGOs play to build resilience at community level in Ambanja district in Madagascar? Finally, how and why mechanisms used by crisis-affected communities and stakeholders have been effective in or hindered responding to climate-related crises in Ambanja district in Madagascar? Through our analysis we contribute to literature on the nature of social capital in building crisis-resilience and the nature and impact of non-state actors (particularly NGOs) in supporting this.

## Methods

### Study setting and population

This study was conducted in Ambanja district located in the North-West Region of Madagascar. The district has 24 municipalities (communes) from which five municipalities were selected for this study as a research area counting a population of 37,784 composed mostly by fishers and farmers (population data collected from the municipalities’ annual reports). The majority of these people depend on fishing and agriculture activities for their daily life [35, 36, 38]. Madagascar, especially Ambanja region, has been exposed multiple times to repeated cyclones with subsequent floods and drought threatening lives and affecting food production and livestock of the population [35, 36, 38]. Ambanja district was chosen for its particularities of being exposed to multiple cyclones more than other regions. Some villages of Ambanja district are affected frequently by floods even in the non-cyclone period due to heavy rain threatening lives, destroying crops and environment including water sources [38, 39]. There are some NGOs that are supporting community members to cope with this difficult situation, with three found to be present during the research. These NGOs have been working in the region for several years helping community members to cope with the challenges of cyclone adverse events. Because of their longstanding presence, we categorise them as “embedded NGOs”.

### Study design, inclusion criteria, and fieldwork team

This is a qualitative study using primary data collected through In-Depth Interviews (IDIs), Focus Group Discussions (FGDs), and Key Informant Interviews (KIIs) in Ambanja district in Madagascar. For IDIs and FGDs, participants were recruited purposively among community members in diverse categories such as farmers, fishers, youth, women’s groups, and school teachers. The choice of participating in either an IDI or FGD was given, in case people were uncomfortable (or unfamiliar) with one on one meetings. For KIIs, participants were selected among local leaders such as the Community Health Workers (CHWs), chief of the villages, chief of basic health centres, and the staff of NGOs that have intervened in cyclone response and accompanying the community to build resilience. The NGOs have been supporting established groups of fishers/farmers and local committees for risk reduction and management in different villages of Ambanja district to reduce the consequences of climate change that they are exposed to. Hence, it was important to understand how they interact with community members to implement their interventions responding to cyclone-related crises.

For IDI and FGD, participants selected were residents of Ambanja district for at least 1 year, aged 18 years old or above, and were able to provide valid consent for the study. For KII, participants were over 18, either local government officials or key staff of the NGOs (four NGOs were involved, their names are not cited here to protect confidentiality). They are NGOs that have intervened in Ambanja district to support communities in their fishing/farming activities or responding to epidemics/outbreaks and/or cyclone preparedness. Participants from these NGOs were able to provide valid consent for the study.

### Data collection, management, and research team

Data were collected from 27 May to 30 September 2024 through IDI, KII, FGD, and from participants in workshops to validate data and collect additional data for clarification. IDI and FGD data were collected from community members while KII data targeted local government officials and key staff from NGOs that had interventions in the field to support community members during cyclone periods. The fieldwork for this research was undertaken by the principal researcher (MKS), a research coordinator (AAR), and three local research assistants (SR, KV, TM) who are fluent in both French and local languages (Malagasy and Sakalava, languages that are most spoken in the region). They all have master’s degrees and are working within the health system at the District Health Office in Ambanja. The research team consisted of six females and three male researchers. No specific relationship with participants was established prior to the start of the research except the fact that junior research assistants and the field research coordinator had regular visits to the study villages for their routine surveillance and monitoring activities.

Most KIIs were conducted by the principal researcher (MKS) for participants who selected French as preferred language for interview. Some IDI were also performed by the principal researcher (MKS) for participants who were comfortable to attend the interview in French. All the FGD and some IDI were conducted by the local research assistants as many people chose Sakalava as their preferred language for the discussion. Participants were invited to a specific place in each village (IDIs, FGDs, and some KIIs) and in the Ambanja city (KIIs) to attend the interview meetings. No one other than the participants were in attendance during the interviews. Data collection was guided by the principle of saturation for IDIs, KIIs, and FGDs starting from the third field visits.

The interview guides (supplementary material 2) were developed by the lead author (MKS), reviewed by co-authors (NS, JS, and SM), then validated by the senior author SM. The research field team was trained on these tools and during this training a simulation was done to pilot test the guides checking its length, clarity and comprehension of questions with the local research team before use for data collection. The duration of IDIs and KIIs was between 35 and 50 min while the FGDs lasted between 50 and 60 min.

Interviews and group discussions were conducted in five villages from five municipalities of Ambanja district (villages A, B, C, D, and E, these villages are anonymised for ethical purposes to protect our participants) and in Ambanja city for KIIs. The research team contacted local mobilisers by phone to prepare the participants for a meeting to conduct interviews or group discussions at specific locations in the villages. All participants reached in the mobilisation accepted to attend the interview meetings. IDI and FGD topic guides collected the same information, but participants were different (based on their preferred format).

Table 1 below shows the size and data sources for KIIs, IDIs, and FGDs.

**Table 1:** Source of information for data collection

Villages	Source of data		
	Key Informant Interviews (KIIs)	In-Depth Interviews (IDIs)	Focus Group Discussions (FGDs)
A	1 (*n* = 1)	1 (*n* = 1)	1 session (*n* = 8)
B	2 (*n* = 2)	3 (*n* = 3)	1 session (*n* = 10)
C	1 (*n* = 1)	1 (*n* = 1)	1 session (*n* = 7)
D	1 (*n* = 1)	3 (*n* = 3)	1 session (*n* = 8)
E	1 (*n* = 1)	2 (*n* = 2)	1 session (*n* = 9)
Ambanja city (stakeholders from NGOs)	10 (*n* = 10)	-	-
Total	*N* = 16 (7 females and 9 males)	*N* = 10 (3 females and 7 males)	*N* = 42 (13 females and 29 males)

The topic guides for KIIs, IDIs, and FGDs (Supplementary material 2) were developed with guidance from a realist-informed framework developed within the overarching project [33]. The questionnaires were framed in way to gather insights on a context in which extreme weather events happened. Hence, questions related to what kind of event and how it affected community members were included as well as questions related to coping mechanisms and interactions between community members and stakeholders to support the response to cyclone-related shocks. These were questions such as: How did you cope with this situation? Were there any actions – by you or others – put in place to solve the issue? What were these actions and what was their purpose? Questions related to local social capital were asked such as: Is there a group of people in your community ready any time to respond to a health/climate related crisis? If yes, how do they work? Do they need external support to work more effectively? Furthermore, questions related to outcomes were included such as: what was the results of your interactions with NGO staff to respond to cyclone crisis? Were your contributions considered by the people who invited you to participate? In your view, were these actions successful? If yes, what made them successful?

Workshops were organised to disseminate the findings and validate the contexts, mechanisms and outcomes (CMO) identified in the research. In addition, they helped generate additional data through discussion and to clarify uncertainties from the primary data such as the reason why people refuse to relocate from their villages even when the situation is dangerous, what can be done to make them more resilient in their villages, and what strategies are needed to maintain environment conservation practices sustainable.

Both interviews and FGDs were recorded using dictaphones and laptops, then were directly transcribed into French by local research assistants and then translated into English by the principal researcher. All the transcripts (in French) were sent to the principal researcher for quality check and approval. The quality check was done on a regular basis during the data collection period in a weekly meeting with the local research assistants. The team went back to field for follow-up questions when any relevant information was missing after quality checking. Some follow-up questions were followed up by phone. In total, three additional physical visits and five phone calls were conducted for follow-up with participants for a total of eight participants.

### Conceptual approach and data analysis

A mix of inductive and deductive thematic analysis was used. Data were coded by the lead author (MKS), reviewed by co-authors (NS, JS, and SM), then validated by the senior author SM. Our data collection was guided primarily by the realist-informed approach considering the principles of Context (C), Mechanism (M), and Outcome (O) (CMO) of community involvement in addressing health and environmental related crises (due to cyclones) by responders [40, 41]. In line with the realist evaluation approach, we first conducted two literature reviews: one of conceptual literature and official guidelines available on engaging communities in crisis-response [33] and another reviewing published and grey literature on community engagement in cyclone response in Madagascar. The literature in both reviews was too limited to identify any specific hypotheses on community engagement mechanisms which might have informed our data collection, but our conceptual review paper was able to construct a framework of the likely factors shaping context, mechanisms and outcomes for community engagement in crisis-response. Therefore, our topic guides were informed by this framework, and we developed questions relating to each of the three CMO categories, with a particular focus on understanding the mechanisms of engagement, how these were shaped by local geographic and village contexts and what observed or reported outcomes they led to.

At data analysis stage, the realist-informed CMO approach was used to extract information about local contexts in which villagers respond to the cyclone and flooding threats, whether and how they engage or collaborate with external actors (government, NGOs), the actions that they take (mechanisms) and the outcomes that result in terms of community engagement and improved health and livelihoods (Excel sheet was used to extract these data). As far as possible we sought to analyse what works and what does not, in what context and how [42]. Starting with observed or reported outcomes, we sought to trace the pathways by which community engagement mechanisms led to these outcomes and how they were shaped by local contexts. To do this, we developed narrative summaries (Supplementary material 3) from the data transcripts using MS Word, from which we derived a series of descriptions of CMO configurations (CMOCs). From our analysis three clusters of explanatory CMO configurations emerged from which three facilitators of change (FOC) categories (which are pathways for resilience) were described which collectively contribute to one overarching theory of change (TOC) (overarching pathway for resilience) on the nature of stakeholders and community engagement that can build local crisis-resilience.

**Fig. 1:**
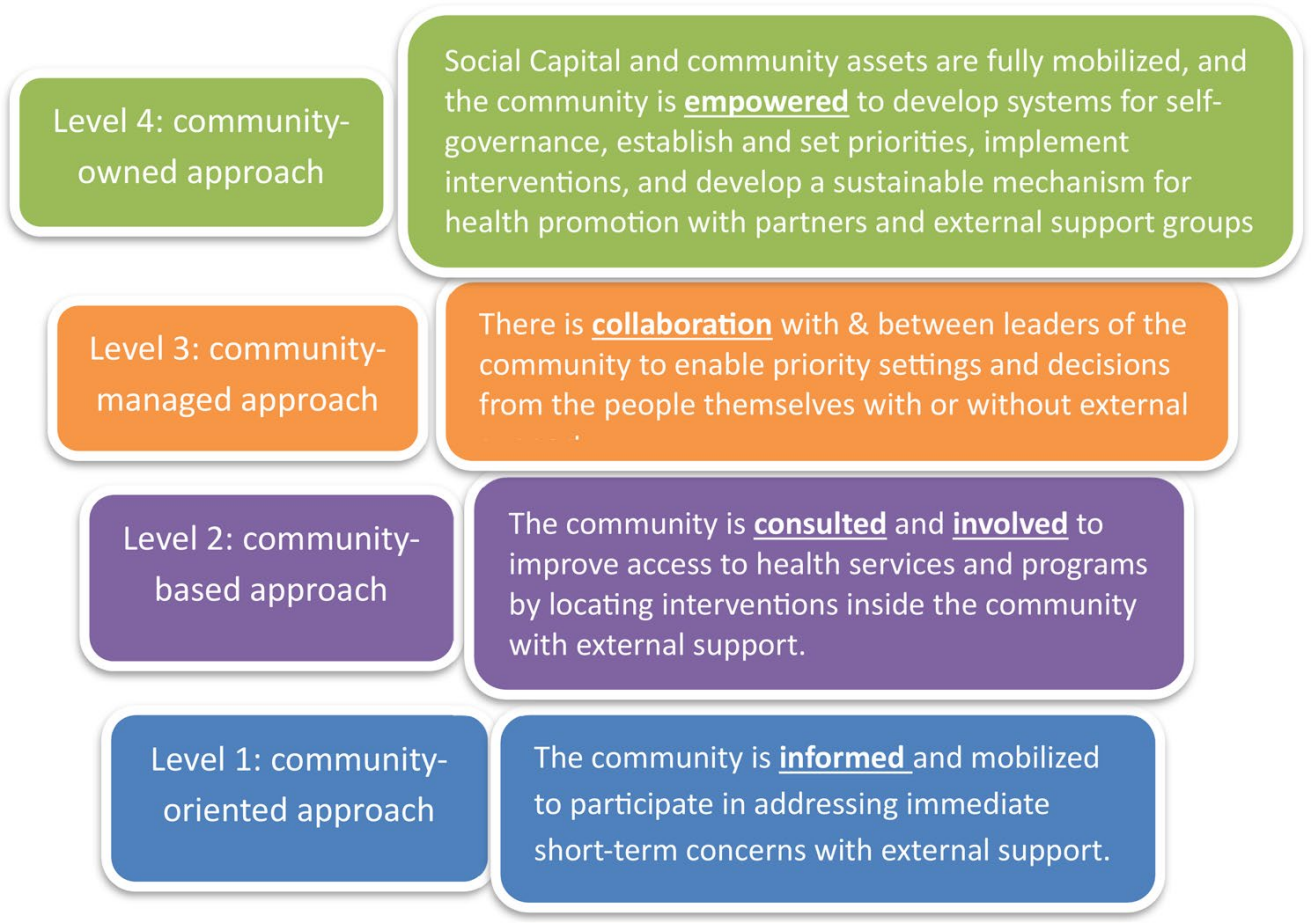
Levels of engagement framework adapted from WHO community engagement and involvement for health improvement [43, 44]

Finally, the World Health Organisation (WHO)’s Community Engagement and Involvement (CEI) framework [43] then guided our assessment of the level of engagement by stakeholders, as seen in the CMOCs, using the five framework elements: Inform, Involve and Consult, Collaborate, and Empower (Fig. 1).

No other themes were relevant for this manuscript. The findings report was guided by COREQ guidelines and a COREQ checklist was filled for the reporting process (Supplementary material 4).

## Results

In total, 10 IDIs (*n* = 10), 16 KIIs (*n* = 16), and 5 FGDs (*n* = 42) were analysed for this study. The specific context for this study is that repeated cyclones in Madagascar significantly impact communities. At the community level, cyclones cause difficult living conditions by destroying crops, houses, roads and other infrastructure. In addition, routine activities that support daily life are stopped during cyclones. Furthermore, each cyclone leaves behind another crisis of floods which exacerbates the difficult situation that people experience. Repeated cyclones led to repeated damage to crops, increasing the risk of hunger in the region. This has also meant that people are unable to set aside sufficient amounts of food to feed themselves during the cyclone periods.

Below, we present the outputs of our analysis that identified specific mechanisms used by both community members and external stakeholders to cope with these cyclones, and their subsequent outcomes. One overarching theory of change was developed that encompasses all identified mechanisms: stakeholders, especially embedded NGOs (i.e. long-term present in communities), who are willing and able to work in equitable partnership with communities can provide a significant contribution to addressing community-identified challenges; through this way of working, they can build trust and contribute to community resilience. This TOC has three underpinning sub-theories that we have called ”facilitators of change” (FOC), each of which is supported by a group of CMOCs. These are shown in Table 2 below.

**Table 2:** TOC, FOCs and their subsequent CMO configurations

Overarching Theory of Change (TOC): overarching pathway for resilience		Stakeholders (particularly embedded NGOs) who support local communities in equitable problem solving can build trust and contribute to building community resilience.	
No	Facilitators of Change (FOCs): sub-pathway (group) for resilience	CMO (Context-Mechanism-Outcome) Configurations	Main outcomes
1	*Timely and trusted risk communication and early warning systems trigger immediate response and ongoing NGO collaboration to save lives (FOC 1).*	*CMOC 1*: NGOs and local authorities train and sensitise communities for preparedness actions (C) and when meteorological office and Bureau National de Gestion de Risque et de Catastrophes (BNGRC) get information about cyclone path and timing (C), they use established, trusted communication channels (well organised infrastructure) to inform the authorities at the district level (M: resources), who liaise with NGO partners and local authorities at the village level to support preparedness actions (M: strategies; reasoning) and the community put the recommendations in practice (M: Actions). This led to reduced death rate during cyclone and increase community resilience (O). *Refence: Supplementary material **3*, *narrative summary 6.* *CMOC 2*: During the cyclone passage (C and trigger), the embedded NGOs and local authorities communicate with community using coloured flags, megaphones, and whistles (M: resources and action), asking people to stay in their houses (M: reasoning), while constantly listening to information on radio about the progress of cyclone (M: action). The supporting NGOs distributed radios to households who did not have them to keep them active in the cyclone monitoring process (M: strategy also action and resource). This led to reduced death rate during cyclone and increase community resilience and trust towards the supporting NGOs and local authorities (O). *Reference: Supplementary material **3*, *narrative summaries 6*, *15* *CMOC 3*: When the cyclone has passed over in the village (C), the embedded NGOs and local authorities communicate with the community using blue flag, whistles, and megaphones (M: resources and action? ), showing that people can now go out of their houses (M: reasoning), but they ask them to continue to follow the communication being shared on the radio (M: action) because the cyclone could still come back and there is likely to be damage caused by the cyclone that needs repair (M: strategy). This continued engagement led to community resilience and trust towards the supporting NGOs and local authorities (O). *Evidence: Supplementary material **3*, *narrative summary 6*	Improvement of health indicators (reduction of death due to cyclone), community resilience, and trust.
2	*Embedded NGOs that understand and work with communities*, *build trust and are able to mobilise local people for joint actions and progress towards shared goals (FOC 2).*	*CMOC 4*: When locally operating NGOs (C) engage with and use local knowledge (M: resource) to inform their interventions (M: reasoning/strategy) then actions taken (e.g. protecting wells) (M: response action) are more effective/sustainable (O) and trust with communities is enhanced (O). *Reference: Supplementary material **3*, *narrative summaries 2*, *14* *CMOC 5*: When NGOs have been active in the local area for a long time (C) they gain knowledge and understanding of the local context (social networks, community structures, communication channels, community needs and preferences, key community leaders etc.) (M: resources; reasoning) which leads to greater trust and greater willingness to collaborate by the community (M: resources, strategy) which triggers joint activities that address community needs and priorities (e.g. riverbank protection; food security) (M: triggers, response actions) leading to reduced flooding and better food stocks and better longer-term resilience (O). *Reference: Supplementary material **3*, *narrative summaries 2*, *6*, *18* *CMOC 6*: After seeing that mangrove forests were destroyed and knowing that this would contribute to reduced productivity of local fisheries (C), NGO 1 approached the communities to discuss with them mangrove restoration (M: reasoning). The NGO engaged local communities in mangrove forest restoration (M: resources and action) in exchange for a donation of useful household objects (M: strategy). This provided community members with the motivation they needed to undertake this work, and this intervention helped them both to increase fish production and decrease the wind pressure on the village (O). *Reference: Supplementary material **3*, *narrative summaries 17*, *18*, *and 19*	Community resilience and trust towards supporting stakeholders
3	*Communities contribute substantial social capital in the form of knowledge*, *social networks and labour that NGOs can harness and support to build crisis resilience (FOC 3).*	*CMOC 7*: When the NGOs saws that people did not have access to clean water in the village B (C), they decided to construct water wells (M: action) to ensure people access clean drinking water (reasoning). However, they did not consult the local communities to understand where and how to construct these wells(strategy). The constructed wells were destroyed by the repeated floods after each cyclone and are no longer usable (O). *Reference: Supplementary material **3*, *narrative summaries 5*, *7* *CMOC 8*: After a cyclone, village C was severely flooded, restricting people’s ability to seek food (C). People living in the neighbouring villages decided to help them (M: reasoning) and brought food helping them to survive during the difficult periods (M: action) using their traditional wooden boats (M: resource). They brought any kind of food that was available (M: strategy and action/resource? ) to help ensure those most affected were able to survive the flooding period (O) even though some of the foods were spoiled and created enteric illness (O). *Reference: Supplementary material **3*, *narrative summary 7* *CMOC 9*: When NGO 4 saw that people in village D were producing less rice due to repeated floods in their rice fields (C), it decided to support the village to address this issue (M: reasoning) by constructing a defence wall alongside the sea (M: action). The NGO provided training to community members and gave them funding and equipment to support and establish local initiatives and restore their agricultural activities (M: strategy and resources). This empowered and helped them to take ownership to solve challenges when they occur (for instance, maintenance of defence walls) at the local level helping them to secure food production (O). *Reference: Supplementary material **3*, *narrative summaries 16*, *22* *CMOC 10*: As the community members in village D experienced the benefit of having a defence wall that protected their rice fields from flooding for five years (C), they expressed a need for the wall to be repaired when the wall was damaged (M: reasoning). In the absence of external support, the community members decided to continue maintaining the damaged parts of the wall with their own local means such as mud (M: action) even though these kept being destroyed by high tides from the sea. They need support from stakeholders with sustainable materials such as cement and they are ready to provide the labour (M: strategy). This shows their level of ownership and the willingness to protect livelihoods production for the village (O). *Reference: Supplementary material 3*, *narrative summaries 16*, *22*	Not using local social capital (local knowledge, social network) can hinder health outcome; community resilience;

The three underpinning sub-theories describing the “facilitators of change” (FOC) are described in detail in the following sections which draw on case data from the narrative summaries in Annex (Supplementary material 3).

### Coordinated risk communication and stakeholders’ collaboration saves lives

#### FOC 1

“*Timely and trusted risk communication and early warning systems trigger immediate response supported by ongoing NGO collaboration to save lives”.*

Ambanja has a well-developed and well-functioning early warning system that helps reduce risk to life. A communication system called Cyclone Early Warning System was established and is managed and coordinated by the National Office of Risk and Disaster Management (Bureau National de Gestion de Risque et de Catastrophes [BNGRC]). Early warning information is communicated via the phone from the Meteorological services to the district and the municipality levels. The municipality liaises directly with the village chief and the local committee of BNGRC. Each village has its own GRC (Gestion de Risque et de Catastrophes) local committee which coordinates actions at the village level. This committee has the role to inform about the immediate risk of cyclone at the village level, allowing locals to start the preparedness process with support from the NGOs (mostly NGO 2 and 3, sometimes NGO 1) on the ground and the GRC local committee.

At village level, the cyclone passage (and level of risk) is communicated using four differently coloured flags (see Supplementary material 3, narrative summary 23 for details). The flags are raised by the village chief at each step of the cyclone event alongside the use of whistles and megaphones by local authorities and the local GRC committee members and continuous communication on the radio. Each household is advised to follow instructions given by the committee and the information on the radio. Radios were distributed to community members who did not have them by NGO-2, to ensure the information reached everyone in real time.

The same NGO 2 in collaboration with the local GRC organised repeat training sessions on cyclone preparedness (reminding people about the flag-warning system) and providing training and materials for protecting riverbanks from flooding, noted that people were committed to spending time with them for training, suggesting that they had trust in the NGOs to provide life-saving support:

*“We are sure that people trust us because they have been with us for more than two full days during the training to prepare for cyclone passage even when we did not have to give money for motivation. They were engaged in the discussion and committed to implement the instructions that we gave them [K-08-M*, *Ambanja town]”.*

The presence of local authorities and NGOs like #2 that have supported cyclone-response for a long time, continuously communicate with local people and offer training and support, increases the trust of people towards these stakeholders.

*“We have seen NGO 1 and 2 supporting us for long time and gave our request to them when we need something. They usually respond and if they are not able to solve the problem*, *they tell us and propose what is possible for them. We really trust them because they try their best to solve the problem [F4-06-M*, *village D]”.*

Observed improvements in preserving life also help build motivation to act on early-warning instructions and a positive perception of the NGOs who support this.

*“We get the communication well in advance and this allows us to prepare ourselves to face the cyclone. Since this programme started*, *no one dies due to cyclone passage. Only some exceptional people who do not respect instructions*, *trying to go to the sea for fishing during cyclone passage. This happens sometimes but it is not very often [F3-04-M*, *village C]”.*

### NGO embeddedness builds trust and longer-term resilience

#### FOC 2

“*Embedded NGOs that understand and work with communities*, *build trust and are able to mobilise local people for joint actions and progress towards shared goals”.*

The embeddedness of NGOs emerged as a key factor facilitating the involvement of community members in response to cyclone crises. Although many NGOs have supported community members in different ways to cope with cyclone-related crises, there are currently only two NGOs on the ground that can be termed “embedded”. These are the NGOs that were present for many years and who were revealed as more responsive to community needs and show greater mutual collaboration and partnership for community resilience.

Embedded NGOs consulted and collaborated more with locals to implement preparedness actions. There were local self-support groups created at the village level by community members with support from the NGO-1, which are called GEC (Group communautaire d’Epargne et de Credit). Members contribute money every month to these groups, and they are supported by NGOs by training in resource and finance management. GECs then lend money to their members that they use to buy food, allowing them to stock up for the cyclone period (when food-production or purchasing is not possible). This collaboration between communities pooling their money and NGOs supporting group and financial management helped community members become more resilient. The money is reimbursed during non-cyclone periods when the members harvest from their gardens. Importantly, while the GEC initiative came from NGO-1, it was the villagers who requested financial management training, and the NGO responded:

*“We have raised awareness to encourage people at the community level to organise themselves as economic self-help groups which was done in different villages. They have asked us to train them in finance management and that is what we do to ensure their committees have sufficient capacity in resource management. We do regular follow-up with them to discuss their challenges and needs [K-02-M*, *Ambanja city*, *NGO 1]”.*

It is this embeddedness and responsiveness that contributes to villagers trusting these NGOs stakeholders and allows long-term collaboration to manage recurring crises.

As well as building trust through their committed presence, embedded NGOs better support local communities, to the extent of their capacity, for long-term resilience by providing resources to protect livelihoods and reduce risk from floods after cyclones. NGO 2 collaborates with locals for joint planning of risk reduction interventions such as planting Napier and trees and placing sandbags on riverbank and the seacoast to prevent erosion. NGO 1 collaborates with and involves communities in protecting and replanting the mangrove forest with the purpose of restoring the environment and increasing fish production. Community members were incentivised to undertake these activities as the NGO 1 provided household items to people involved in return for the labour they offered. NGO 1 also supported fishermen to respect a seasonal fishing closure, as a way of increasing productivity of the fishery. In addition, community members were able to request needed tools and materials from these embedded NGOs.

*“When we saw that wells constructed by [NGO 4] were not providing drinkable water*, *we have requested the NGO 2 to support us getting clean water. They have promised to supply water to our village from the neighbouring village. They took one of us to identify households that need private meters and those who will use public meters. We hope that they will start this work in the next few months [F1-01-F*, *village A]”.*

*“[NGO 1] has asked us to work together to restore our mangrove and we were happy to do this because they have explained to us its importance. As compensation*, *they gave us household’s cooking materials such plates*, *pots*, *ladles*, *spoons*, *bowls in return for us replanting 5 hectares of mangroves [F1-07-M*, *village A]”.*

The seasonal closures are now managed by local leaders, and some community members sought to adapt these to meet their food needs. As the main sources of incomes for most villages are fishing and agriculture, people suggested to be allowed to fish for a few hours daily for their food provision during seasonal closure periods to help them manage food stocks for cyclone preparedness.

*“The closure period helps us a lot because when we start fishing after closure*, *we see significant increase of our production. However*, *we do not have sufficient stock of food and people feel obliged to go to the sea for fishing sometimes when their food stock is over during cyclone. We have asked our authorities to allow us to fish just the quantity that we can eat [I-05-M*, *village D]”.*

Although NGO 1 made efforts to restore mangrove forests with the purpose of ecosystem restoration, there were additional benefits stated by the community members in village A. They reported that the restored mangrove protected their houses against the wind during cyclones:

“*We do not have a big problem with wind pressure during cyclone in our village because the mangrove around us protects us against the wind [I-01-F*, *village A]”.*

Embedded NGOs were able to support long-term resilience building by understanding local needs and initiating major projects (like mangrove restoration) to address them, but they also worked closely with local leaders and community members to plan and implement joint activities, and respond to community requests (for training, resources) and build capacity for local ownership and management (as seen in the GEC and fishing-closure examples).

### Use of local social capital: local knowledge and social networks

#### FOC 3

“*Communities contribute substantial social capital in the form of knowledge*, *social networks*, *and labour that NGOs can harness and support to build crisis resilience”.*

This study found that the community has important local knowledge – a form of social capital – that stakeholders should make good use of to inform their interventions in the response to cyclone-related crises. Communities suffer a cascade of crises originating from a cyclone which subsequently leads to flooding then infectious diseases (outbreak or not depending on the defined disease threshold). In our study villages, most cyclones led to flooding, and communities developed specific strategies to cope, based on experience and knowledge.

Their strategies are the following. First, they adapt their house construction (on stilts) which helps people to remain in their houses even when the village is flooded. The houses are constructed on long pillars of 1.5–2 m. Second, communities pool resources to help each other. Some use traditional wooden boat to move around fetching forest food (yams and casava) and firewood during the flooding period; young people organise themselves in groups of 2 to 3 to search for firewood for a group of households. Third, knowing when daily tidal floods came (usually between 14.00 and 17.00) helped people plan and prepare by ensuring children and other vulnerable people were taken care of to avoid drowning; they also ensure that everyone has eaten lunch before that time as no household activities can be done during flooding. Fourth, cow-drawn carts are used to fetch water from the neighbouring village located 3–5 km away allowing people to get potable water. This allowed people to deal with water stress in the absence of protected water sources in the villages.

It is very important that local knowledge is used to help plan future interventions – when it is ignored, interventions are less impactful and resilience is less likely to be built. In village B, local community knowledge and norms were not considered, leading to resources being wasted by an unnamed NGO who came in to build water wells that were not flood proof and therefore repeatedly flooded following cyclones. Hence, these wells were destroyed and are now not useful. The community members know how these resources could be constructed to be resistant to floods, but they were not consulted and now they have no water resources to use and they use either water from the river during non-cyclone periods or from floods during cyclone periods exposing them to risk of enteric infectious diseases.

It is important to note that most villages do not have latrines because of lack of capacity due to the burden imposed by floods during cyclone periods. The latrines construction in the areas prone to floods in this region will need resources to protect them against the flooding events to avoid overflow, which may be expensive. As they lack these resources, they have opted not to construct unprotected latrines. Instead, community members clearly explained that latrine construction should follow the same model as their houses and this is what needs to be funded to be successful.

*“We do not construct latrines because they need to be made in a special way especially for this village that has severe floods during cyclone periods. They need to be at a height of 2 m with stairs and [we] need sufficient means [support] in term of cement, rebar, and manpower. We are not able to find that unless we get support from the government or NGOs [I-06-M, village B]”*.

During cyclone events, the difficulties that some community members experienced led to supportive actions by their neighbours. After severe flooding in Village C, for example, people from the neighbouring village used their traditional wooden boats to reach Village C, bringing food to people who are trapped and in need. They brought any kind of food that was available to them including dead animals and green bananas found in the flood water which led to enteric diseases for some people. Even though some of the food was not fit for consumption, this shows a strong willingness and solidarity to support people in need. An informal social network in the community was spontaneously formed with local people volunteering to support affected communities: people bringing food to those who are stuck in the flooded villages and within the affected villages, people who have solid resistant houses accommodating those who live in unsafe places and in fragile houses that cannot resist the cyclone wind.

*“Here, people agree to receive others in their homes during the passage of the cyclone or during the flood. Yes, that is what we always do. People agree to receive others to accommodate them, and they return back to their homes a week after the cyclone has passed, so we are sure that the danger is over (F5-06-F, village B)”*.

It was noted that this community social network is not a formal structure and yet it is an opportunity that stakeholders can consolidate and use to reach and support local communities during difficult periods.

Another example illustrates how local ownership of an NGO-initiated intervention can continue after initial NGO support ceases. In 2015, Village D was suffering regular crop destruction due to repeated floodings of their rice field plantations. NGO-4 became aware of this and approached the village to help them by constructing a defence wall to protect their livelihood. The construction work was done jointly with the community because the NGO provided technicians and resources while community members participated by providing labour. In return the NGO created a local committee of volunteers from community and provided them with equipment such fishing nets, plank cutting machine, machine to husk rice allowing them to create local initiatives for the village that were accessible to all inhabitants of the village. All benefits from these activities were used to solve the common challenges of the village. This was a success ensuring protection until 2020, when the wall started presenting weak points allowing sea water to seep through, bringing back flooding of rice plantations after 5 years of protection. By then, NGO-4 was no longer present, and no other stakeholder took over the initiative. Instead, community members have kept maintaining destroyed walls using their own, limited local means (mud) and working manually. The lack of external support to provide them with long-term resilient materials for effective repair is a challenge because their efforts are always destroyed at every episode of cyclone or flooding. Nevertheless, they are committed to repairing as best they can because they experienced the benefits offered by the defence wall.

## Discussion

We found that there is little consistent support to people in our study villages in Madagascar who continuously face extreme difficulties because of cyclones. Therefore, people have developed their own strategies to build their resilience and improve health outcomes during cyclones, though the impact was often limited because of lack of external resources. The main consistent support came from two embedded NGOs (with a long-term presence) who work with communities and contribute both to immediate health outcomes (keeping people alive during the passage of a cyclone) and longer-term support. Three distinct facilitating pathways to better crisis-response were identified. First, coordinated, timely and trusted risk communication and stakeholder collaboration for cyclone-protection saves lives. Second, NGO embeddedness builds trust and longer-term resilience. Third, local knowledge and social solidarity networks constitute important social capital that must be engaged by NGOs and other stakeholders to build resilience.

Two important key challenges emerged. First, most NGOs (and government stakeholders) only provide short-term support and focus only on life-saving interventions during the cyclone passage, ignoring longer term crises such as flooding, environmental degradation and related disease outbreaks. Tackling these wider, persistent challenges would greatly help in building long-term resilience. Second, some NGOs (especially those who only have a short-term presence) fail to recognise or use local knowledge (social capital), leading to wasting resources on non-sustainable interventions (such as non-floodproof wells). These issues were observed across all the villages covered by this study.

Considering how NGOs engage communities and their social capital in crisis-response, according to the CEI stages in the WHO framework, reveals the differences in how stakeholders (particularly NGOs) operate and illuminates both the negative consequences of poor engagement and how these challenges can be overcome.

It was clear that NGO support to bolster the government’s cyclone warning systems clearly saves lives. This is akin to the “inform” level of CEI framework. While important, merely informing people about impending cyclones does not empower them to become more resilient to these adverse events.

Local knowledge and existing community capacity are key social capital assets. When these are not adequately incorporated into programme design, NGOs may inadvertently compromise resource efficiency and intervention sustainability. Our study reveals a concerning pattern: whilst a few embedded NGOs pursue long-term, resilience-orientated work, most NGOs in our study appear to implement interventions without systematically consulting or involving communities (the second framework stage) about their insights and needs, potentially leading to suboptimal resource allocation and missed opportunities for enhanced effectiveness. In Village B, for example, NGO-constructed water wells are now non-functional due to flood damage because programme designers did not consult residents about flood-resistant construction methods, effectively negating the investment whilst leaving the community vulnerable to enteric diseases as residents resort to consuming flood water during cyclone periods. In some of our case study villages where external support was lacking or misguided the presences and importance of social capital was revealed. In Village C, and elsewhere, there were notable solidarity networks in existence, where community members provide mutual support through food provision (albeit limited by poverty) during crises. These indigenous support systems represent valuable social capital that most of the NGOs in our study appear to underutilise, perhaps favouring their own standard practices – as has been seen elsewhere – rather than building upon established, community-based approaches that have demonstrated resilience over time [33, 43]. This adds another concern to cautions already expressed in the literature on non-state provision of public goods, which suggests that NGOs and other non-state stakeholders can distort accountability and perpetuate inequities when they control responses on their own terms [30].

Our findings on the final stages of the CEI framework, however, add depth and nuance to existing literature on nonstate provision of public goods. The most positive examples of building community-level *resilience* came from long-term support provided by embedded NGOs who consciously sought to collaborate and empower local leaders and community members – attributes of the third and fourth stages of the CEI framework. Literature from elsewhere has also shown that this is key to ensure successful behaviour change [33, 43, 45], and community dialogue to reinforce this as part of preparedness planning was found to be valuable to contribute to community resilience [33, 43]. Our research shows that where NGOs were embedded in communities, they had a deeper understanding of social capital and more reciprocal, equitable and trusted relationships with communities. We argue that where these types of relationships can be built, then NGOs can play a critical and positive role in safeguarding public goods and bolstering social capital so that its impact can be maximised in very low-resource contexts that could otherwise limit it (e.g. in food provision). Our findings also give depth to the social capital scholarship most of which is not from low-income settings. While key scholars suggest that social capital is the most important enabler of resilience-building [26, 27], our findings show that in the face of absolute poverty social capital is inevitably limited and external support is necessary for it to have its fullest impact. For example, while people spontaneously formed groups to provide food to flooded villagers, they had no suitable food to give – therefore they were able to prevent people from starving but not from falling sick. External support could have supported their actions by providing healthy food to share. Taken together with our findings on non-state actors, we argue that this role of bolstering social capital in resource-poor settings is best taken on by embedded NGOs.

As work by the World Resources Institute has shown, resilience-oriented programmes do not necessarily require substantial financial investment yet can yield significant returns through long-term sustainable development outcomes [46]. Our findings reveal significant low-cost opportunities focused on enhancing community-centred approaches to resilience programming, with important implications for both policy and practice. Our central finding is that NGOs should ensure their modus operandi enable systematic integration of local knowledge and community representation in co-created planning and implementation protocols and processes. This should include ongoing monitoring of implemented actions in partnership with communities to identify areas requiring modification or improvement. For example, communities identified that locally imposed policies on fishing period closures required modification to enable the flexibility to respond to community food needs while retaining cyclone-protective mangroves. Specific disaster-preparedness and response actions were also identified in relation to health information such as water boiling protocols or water filtration at flooded wells which should be systematically integrated into Community Early Warning Systems (CEWS) as part of preparedness planning.

Second, although with greater resource implications, NGOs should commit to long-term (embedded) presence in communities. This will require a funding and policy environment that enables NGOs to make long term commitments to regions and communities and incentivises them to respond to community needs. This could lead to larger numbers of embedded NGOs able to better support communities and bolster their existing social capital to become more resilient.

Finally, a number of specific beneficial interventions can be identified through our study. Unexpected positive effects were seen in the work of NGOs who were initially focused on a narrower goal than resilience-building. For example, NGO 1 initiated work on mangrove restoration with the purpose of increasing the productivity of local fisheries and contributing to ecosystem restoration. However, the community members reported wider benefits including decreased cyclone wind-speeds with less damage to village houses and the floods being less severe in the village where mangrove was restored. This suggests that there are certain highly strategic environmental interventions that have a critical impact on resilience-building and should be expanded and more intentionally built into programme planning whereby the long-term resilience impacts of environmental interventions are considered.

During the research, communities themselves also identified longer-term interventions (such as flooding-proof wells and latrines; cyclone-resilient flooding defence walls) that they regarded as necessary to build cyclone resilience in their villages. Yet, most NGOs intervening in the region did not have these actions on their agenda of priorities and local government agencies typically were not interested in local suggestions. Wider literature suggests specific interventions that could address community-identified concerns for clean water. These include expanding access to water filtering and water treatment technologies that were implemented in Velondriake region during cyclone Haruna in 2013 [47, 48]. The filters “Family Life Straws” (with capacity of filtering 10 L per hour) that were set at flooded, contaminated wells to filter water for people before use [47, 48] was a promising initiative that could be replicated to other regions, however since they rely on foreign donations they are not sustainable and do not build long-term resilience.

This study had several limitations as it was conducted in Ambanja district which is a large district of twenty four municipalities. Due to logistical challenges, we limited our sampling to five municipalities selecting one village per municipality. It is possible that data may not be representative of the whole district. However, to ensure that we collected accurate data, we selected municipalities (and villages) which are usually more affected by cyclones in the district. Courtesy bias related to hierarchy was resolved by the fact that the junior research assistants, even though they work at the health district office, usually talk to local people in the villages during their supervision activities and people feel open speaking with them frequently about their negative experiences.

## Conclusions

Communities in five villages within the Ambanja region, Madagascar, face significant cyclone-related challenges including floods, enteric infectious diseases, and water insecurity. This study identifies three main pathways that enable communities to develop resilience to these challenges. First, timely risk communication from trusted stakeholders contributes to resilience and can be life-saving. Second, the presence of embedded NGOs plays a pivotal role in mobilising communities, gaining trust and delivering the long-term partnerships and interventions which are essential for addressing vulnerability to cyclone and building sustained resilience. Finally, working with and supporting the existing community social capital (including local knowledge, labour and solidarity networks) present in all five villages, is key to success in responding to cyclone-related crises and contributes to local resilience building. The study identified two fundamental challenges that need attention. Most importantly, the cyclone crisis response is dominated by short-term interventions by organisations that do not have long term relationships with communities (often through emergency humanitarian agencies) with only a few NGOs engaged in long-term interventions. This limits resilience to mere survival but do not contribute to the development of true resilience which would lead to communities being able to adapt and thrive. Additionally, local knowledge and social norms were frequently ignored by external stakeholders responding to cyclones and this led to inefficiencies and interventions that did not produce or sustain the desired results. This was clearly exemplified by water and sanitation facilities that were rapidly destroyed because the external stakeholders failed to incorporate community insights about flood-resistant design. Considerable adaptive capacity and strong social solidarity have been seen at the community level; they are limited only by material poverty. These local resources need to be utilised and bolstered by responders to build sustainable resilience. Longer-term interventions and consistent support that focuses explicitly on building resilience rather than purely responding to crises, must be a priority, if we are to help building resilient communities.

## Supplementary Information

Below is the link to the electronic supplementary material.

Supplementary Material 1: Abstract, French version.

Supplementary Material 2: Topic Guides.

Supplementary Material 3: Data Presentation: Narrative Summaries.

Supplementary Material 4: COREQ Checklist.

## Data Availability

All data used in the analysis of this manuscript are available in the Supplementary material 3: Data presentation: Narrative Summaries.
