# Supplementary material for: The role of embedded Non-Governmental Organisations and other stakeholders in building resilience to cyclone-related crises in Madagascar: a qualitative study
Source: BMC Glob Public Health. 2026 Jul 9;4:66. doi: 10.1186/s44263-026-00300-y (PMC13348428; doi:10.1186/s44263-026-00300-y)
Supplement: Supplementary file 1 — Supplementary Material 1: Abstract, French version. [file 44263_2026_300_MOESM1_ESM.pdf]

This translation in French was submitted by the authors, and we reproduce it as supplied. It has not been peer reviewed. Our editorial processes have only been applied to the original abstract in English, which should serve as reference for this article. This translated abstract is published under the same license as the article.

Résumé

Contexte : Madagascar est un pays qui a été gravement affecté par le changement climatique, avec un impact particulier sur la santé de la population, le bien-être et les moyens de subsistance. Le pays est sujet aux cyclones, les inondations étant des conséquences immédiates provoquant une cascade de crises. Cette étude vise à comprendre comment les parties prenantes (États et Organisations Non Gouvernementales) interagissent avec les communautés pour répondre aux chocs liés au cyclone, comment les communautés elles-mêmes répondent, et quels facteurs facilitent ou entravent ces partenariats.

Méthodes : Cette étude qualitative a collecté des données par des entretiens individuels approfondis auprès de membres de la communauté, des entretiens avec des informateurs clés auprès des parties prenantes, et des discussions de groupes de discussion auprès de membres de la communauté du district d'Ambanja à Madagascar. La collecte des données a été guidée par une approche réaliste qui recueille des données spécifiques au contexte, aux mécanismes et aux résultats de l'engagement et de l'implication communautaire pour la réponse aux crises liées aux cyclones. Les données étaient présentées sous forme de résumés narratifs avant une analyse approfondie du cadre.

Résultats : Cette étude identifie trois voies permettant la résilience communautaire. Premièrement, une communication rapide d'alerte précoce sur les cyclones provenant des parties prenantes de confiance, des acteurs locaux et des Organisations Non Gouvernementales (ONG) soutenantes, facilite l'implication communautaire et sauve des vies humaines lors d'événements cycloniques. Deuxièmement, les ONG intégrées engagées à travailler avec les communautés jouent un rôle clé dans la création de la confiance et la mise en œuvre conjointe d'interventions à long terme, essentielles à une résilience durable. Troisièmement, le capital social communautaire existant — y compris les réseaux locaux gardiens de connaissances, de travail et de solidarité — représente des ressources vitales mais qui sont sous-utilisées pour une conception efficace des interventions. Cependant, deux défis fondamentaux sapent systématiquement les efforts de renforcement de la résilience. Plus important, la prédominance des partenariats à court terme signifie que seules quelques ONG (très peu) s'engagent dans des interventions à long terme, laissant d'importants besoins communautaires non satisfaits et limitant la résilience à la survie plutôt qu'à des niveaux propices à la pérennisation. De plus, ignorer les connaissances locales et les normes sociales conduit au gaspillage de ressources par des interventions non durables, illustré par des installations d'eau rapidement détruites parce que les parties prenantes n'ont pas intégré les connaissances communautaires dans la conception pour les modèles résistants aux inondations.

Conclusions : Bien que les communautés possèdent une capacité d'adaptation considérable et une forte solidarité sociale, une plus grande résilience nécessite un engagement institutionnel soutenu envers un engagement et un soutien à plus long terme pour répondre aux besoins de la communauté. Cela nécessite une intégration systématique des connaissances et réseaux

communautaires existants, plutôt qu'une dépendance continue à des interventions à court terme conçues de l'extérieur, qui ne s'appuient ni sur les forces locales ni ne répondent aux vulnérabilités sous-jacentes.
