# Supplementary material for: The role of embedded Non-Governmental Organisations and other stakeholders in building resilience to cyclone-related crises in Madagascar: a qualitative study
Source: BMC Glob Public Health. 2026 Jul 9;4:66. doi: 10.1186/s44263-026-00300-y (PMC13348428; doi:10.1186/s44263-026-00300-y)
Supplement: Supplementary file 2 — Supplementary Material 2: Topic Guides. [file 44263_2026_300_MOESM2_ESM.pdf]

Supplementary File 2: COREQ checklist

Consolidated criteria for reporting qualitative studies (COREQ): 32-item checklist

Item No	Guide Questions/Description	Reported on Page #
Domain 1: Research team and reflexivity		
Personal Characteristics		
1. Interviewer/ facilitator	Which author/s conducted the interview or focus group?<br>The lead author (MKS) supported by local team of Research Assistants (SR, KV, TM)	Pg 7
2. Credentials	What were the researcher's credentials? E.g., PhD, MD<br>The authors have the following credentials: MKS (MD, MSc), VM (MD), NS (PhD), JS (PhD), SM (PhD); while junior research assistants who supported data collection have the following credentials: RS (MSc), KV (MSc), TM (MSc).	Pg 1, Pg 7
3. Occupation	What was their occupation at the time of the study?<br>MKS, NS, JS, and SM are researchers affiliated with the LSHTM; VM is medical doctor who also doing research as independent. The junior research assistants are part of diseases surveillance team at the District Health Office in Ambanja/Madagascar.	Pg 1, Pg 7
4. Gender	Was the researcher male or female?<br>The research team was a mix of females (NS, JS, SM, SR, KV, and AAR) and males (MKS, VM, and TM).	Pg 7
5. Experience and training	What experience or training did the researcher have?<br>The researchers have sufficient experience and the needed training to conduct research.	Pg 1, Pg 7
**Relationship with participants**		
6. Relationship established	Was a relationship established prior to study commencement?<br>No specific relationship with participants was established prior to the start of the research except the fact that junior research assistants and the filed research coordinator had regular visits to the concerned villages for their usual surveillance and monitoring activities.	Pg 7
7. Participant knowledge of the interviewer	What did the participants know about the researcher? e.g. personal goals, reasons for doing the research? => Same as above	Pg 7

Item No	Guide Questions/Description	Reported on Page #
8. Interviewer characteristics	What characteristics were reported about the interviewer/facilitator? e.g. Bias, assumptions, reasons and interests in the research topic => Courtesy bias related to hierarchy for junior research assistants.	Pg 25
**Domain 2: study design**		
**Theoretical framework**		
9. Methodological orientation and Theory	What methodological orientation was stated to underpin the study? => Thematic analysis (mixed inductive and deductive analysis) guided by Realist-informed approach and WHO CEI Framework	Pg 9
**Participant selection**		
10. Sampling	How were participants selected? e.g., purposive, convenience, consecutive, snowball => Purposive sampling was applied for this study	Pg 7
11. Method of approach	How were participants approached? e.g., face-to-face, telephone, mail, email => participants were contacted by phone call to schedule the day and time of interview, then interviews were conducted face-to-face (IDIs, KIIs, and FGDs). Face-to-face meeting was also done by a local mobiliser per village to prepare the participants prior to the real interview meeting	Pg 7
12. Sample size	How many participants were in the study? => A total of 68 participants attended the study interviews for IDIs, KIIs, and FGDs.	Pg 8
13. Non-participation Setting	How many people refused to participate or dropped out? Reasons? None	Pg 8
14. Setting of data collection	Where was the data collected? e.g., home, clinic, workplace A specific place was chosen in each village and in the city to conduct the interview.	Pg 7
15. Presence of nonparticipants	Was anyone else present besides the participants and researchers? None	Pg 7
16. Description of sample	What are the important characteristics of the sample? e.g. demographic data, date => Participants were recruited purposively among community members in diverse categories such as farmers, fishers, youth, women's groups, and school teachers (IDIs and FGDs). For KIIs, participants were selected among local leaders such as the Community Health Workers (CHWs), chief of the villages, chief of basic health centres, and the staff of NGOs that have intervened in	Pg 7

Item No	Guide Questions/Description	Reported on Page #
	cyclone response and accompanying the community to build resilience. Should be adult who have lived in the area for at least 1 year or more.	
Data collection		
17. Interview guide	Were questions, prompts, and guides provided by the authors? Was it pilot tested? => The interview guides were developed by the lead author (MKS), reviewed by co-authors (NS, JS, and SM), then validated by the senior author SM. The research filed team was trained on these tools and during this training a simulation was done to pilot test the guides checking its length, clarity and comprehension of questions with local research team before its implantation for data collection.	Pg 7
18. Repeat interviews	Were repeat interviews carried out? If yes, how many? Not repeat interviews as such but some follow up questions for clarifications were asked to some participants (total 8 for the 5 villages)	Pg 9
19. Audio/visual recording	Did the research use audio or visual recording to collect the data? Audio recording was done during data collection, then transcription was done from the audio	Pg 9
20. Field notes	Were field notes made during and/or after the interview or focus group? None. All interviews were audio recorded then transcribed after.	Pg 9
21. Duration	What was the duration of the interviews or focus group? The duration of IDIs and KIIs was between 35 -50 minutes while the FGDs lasted between 50 – 60 minutes.	Pg 7
22. Data saturation	Was data saturation discussed? => data collection was guided by saturation principle	Pg 8
23. Transcripts returned	Were transcripts returned to participants for comment and/or correction? => Validation workshops were organised with participants to discuss and validate the results from the transcripts	Pg 8
Domain 3: analysis and findings		
Data analysis		
24. Number of data coders	How many data coders coded the data? => data were coded by the lead author (MKS), reviewed by co-authors (NS, JS, and SM), then validated by the senior author SM.	Pg 9
25. Description of the coding tree	Did the authors provide a description of the coding tree? => None, from transcripts, CMOs were developed then narrative summaries which constitute the sources of the findings.	Pg 9 - 10

Item No	Guide Questions/Description	Reported on Page #
26. Derivation of themes	Were themes identified in advance or derived from the data? Themes were identified inductively from data, but topic guides were developed from the research framework	Pg 9
27. Software	What software, if applicable, was used to manage the data? We used Excel for coding and MS Word to develop narrative summaries.	Pg 10
28. Participant checking	Did participants provide feedback on the findings? Validation workshops were organised with participants to discuss and validate the results	Pg 8
**Reporting**		
29. Quotations presented	Were participant quotations presented to illustrate the themes/findings? Was each quotation identified? e.g., participant number Quotations are included in the manuscript for illustration with participant ID for each.	Pg 15-19
30. Data and findings consistent	Was there consistency between the data presented and the findings? Findings were derived from data (presented as narrative summaries in a supporting file, S1) after thematic analysis.	Pg 11 - 20
31. Clarity of major themes	Were major themes clearly presented in the findings? => major themes presented in three sub-theories of changes	Pg 11 - 19
32. Clarity of minor themes	Is there a description of diverse cases or a discussion of minor themes? => None	Pg 11
