# Supplementary material for: The role of embedded Non-Governmental Organisations and other stakeholders in building resilience to cyclone-related crises in Madagascar: a qualitative study
Source: BMC Glob Public Health. 2026 Jul 9;4:66. doi: 10.1186/s44263-026-00300-y (PMC13348428; doi:10.1186/s44263-026-00300-y)
Supplement: Supplementary file 3 — Supplementary Material 3: Data Presentation: Narrative Summaries. [file 44263_2026_300_MOESM3_ESM.pdf]

Supplementary file 1 (S1)

Data presentation: Narrative Summaries

We have analysed and presented our data by Narrative Summaries as described below from the five villages. All the villages were anonymised and coded as A, B, C, D, and E for ethical considerations to protect our participants. The names of NGOs from which participants were selected were also anonymised and coded as NGO 1, 2, 3, …

<u>Village A</u>

Narrative Summary 1

Inhabitants of village A experienced serious challenges of accessing clean drinking water because the water wells constructed in their village produce dirty and salty water that they can use only to wash cloths. The water has a roux colour that can be seen when the water is both in the well and in the bucket. These wells were constructed by the NGO 5 long time ago without consulting the local community members and were not maintained and the NGO that constructed them did not come back to monitor the quality of water that the wells produce. Some wells have pumps that are no more functional. In total six wells were constructed and none of them produce usable water. *"NGO 5 built wells for us but unfortunately it didn't work because the water is not drinkable [F1-03-F, village A]"*. The village inhabitants said their preference would be to get pipe water from the neighboring village. They usually travel a distance of 5 km every day to this neighboring village to fetch water that they use for drinking and cooking food. They monitor the tide before travelling to fetch water because they cannot go during the high tide which occurs every day between 15:00 – 17:00 and sometimes early morning. They usually buy this water at a price of 1,300 – 1,500 Ariary per jerrycan of 20 liters. *"We are forced to buy water in the neighboring villages at 1500Ar for a 20-liter can of water [F1-07-F, village A]"*. During cyclone periods, it become difficult for them to fetch water because the village is usually flooded, and this situation obliged them to use flooding water to drink and cook food.

Narrative Summary 2

The NGO 2 came to village A to discuss with the community members about availing clean drinking water in the village responding to the request from the community. One volunteer from the village was responsible of collecting information about people who were willing to get meters at their households in the village. At the time we were collecting data, thirty people had registered already to get the meters in their houses, and the registration was still ongoing. Water was to be drawn from the neighbouring village located at 5 km and the work was planned to start in the next few months. There was a discussion between the NGO 2 and community members about their local contribution that led to an agreement for two categories of people: those who need private meters in their households will be responsible for maintenance and will pay a one-off amount of 60,000 – 100,000 Ariary and those who want to access water at the public stand will pay 50 Ariary per jerrycan of 20 liters. *"As agreed, there will be two types of standpipes: Public standpipes and private standpipes. For those who have applied for a private meter in their name and those who use public standpipes will pay 50 Ariary for a 20 liters can of water. So, with 500 Ariary we will have 10 cans while now we buy 1000 Ariary a single 20liter can of water. The right of a private meter is 60,000 to 100,000 Ariary. Those who have chosen private standpipes will ensure the safety and maintenance of their standpipes [F1-03-F, village A]"*. Community members found this useful because they usually pay water at a high price of more than 1000 Ariary per jerrycan of 20 liters.

Village B

Narrative Summary 3

Inhabitants of village B prepare themselves to face the floods from upcoming cyclone when they get the communication from authorities. They usually arrange their traditional wooden boats in advance and tie them to their houses. Because, their village is always flooded during each cyclone, they ensure that they have the boats ready before cyclone hit starts. This is the only mean that they have available to allow them moving around when the village is flooded. *"So, when the water level rises and our village is flooded, these boats allow us to move around and go anywhere. That is to say, even if we go to the fields, we have to use a boat because all the surrounding places are filled with water[F5-02-M, village B]".* This has been in their usual practices. After getting communication about passage of cyclone every household tie the wooden boat to the house and prepare a sufficient stock of food to protect the family from cyclone issues. They also reinforce their houses to protect them again the wind using ropes that they tie to four wood sticks in the four corners of the house. *"Each one reinforces his house and ties his boat near his house. We are doing all this to prepare and protect our families from the arrival of a cyclone. To reinforce our houses, we tie ropes on each side of the roof. Then we attach pieces of sticks (about 1 meter long) to each end of these ropes. Then we push the sticks well into the ground. All this to prevent the roof from being taken away by the wind [F5-06-M, village B]"*. In the village, people with houses of poor conditions (fragile) are accommodated by their neighbours who have strong houses to protect them during the difficult periods of cyclone. They also put sandbags on the roof of their houses to protect them against the wind during cyclone hit. These ideas came from their own initiative as they have suffered for long time from repeated cyclone hit and the NGOs came to support this initiative. *"Since we are often confronted with natural disasters, we have been thinking about what we need to do to protect our lives. And the training we received from these NGOs also reinforced what we already know [F5-03-M, village B]"*.

Narrative Summary 4

In village B, people prepare themselves before the arrival of cyclone by preparing sufficient stock of food to cover the period of cyclone. However, the stock of food for different households in the village is not usually sufficient and this puts people in difficult situation as the village is always flooded during cyclone hit. The floods blocked the movement of community members making them unable to look for food and fetch water when their stock is over. When they do not have food while the flooding water last long going until three months in the village, they try to find anything brought by the flooding water such as green bananas and died animals to eat and survive. *"So, in order not to starve when there is no food such as rice for example, we eat bananas that are still green. Sometimes bananas are not enough either because sometimes our village is flooded for three months. Everyone tries to pick up the things that can be eaten brought by the water like bananas and died animals etc... In general, the water starts to disappear from March and everyone can go to the fields, go to other villages, or go to Ambanja to look for food [F5-04-M, village B]"*. Most of time, people stayed blocked in the village from November to March, waiting the flooding water to dry before staring any movement for their usual activities. During the flooding time, people lived in difficult situation because they spent more time with their feet in water on top of food scarcity. *"It is very annoying or even unbearable to have your feet always in the water during the flood. For example, my house is still flooded since the passage of cyclone GAFILO, and during the flood my family and I always have our feet in the water from morning to night. The only time we are not in the water is when we sleep on our beds. It's very uncomfortable, but that's what we experience when it's the cyclone season [F5-06-M, village B]"*.

Narrative Summary 5

In village B, community members had challenges accessing drinking water during cyclone periods. The village has no protected water source and people fetch from the Sambirano river. Only few people have tanks to collect rain water that they use to drink and cook food. The majority of the local population do not have means to collect rain water. During cyclone periods, the village is flooded and people cannot move from their houses. This obliged them to use the flooding water to drink and cook food. This flooding water in the village is always contaminated as people use their immediate environment to poo and urinate, they do not have latrines. Most of time, they usually see feces in the flooding water, but they use it because they do not have any other choice. People are aware that the flooding water is not good for health because some of their leaders sensitise them about this. *"We also make the inhabitants aware of the risk of diseases during the flood because all the garbage and dirt from the villages in the upstream part ends up here. The water that floods our village is very dirty, so we always try to convince the inhabitants to always use rainwater for drinking water and food preparation [F5-07-M, village B]"*. Long time ago, water wells were constructed in the village and some of them produced water of good quality before their destruction. The challenge is that the wells were constructed without consulting the local community and did not respect the local norms to allow them resisting against floods. Due to the repetition of floods, these wells are now all destroyed. Most of them are sunk in the soil and cannot be used. The local population thought that these wells could be constructed at a height of 1.5 to 2 meters to resist the multiple floods during cyclone periods, but they were not consulted before the implementation of the project. One well is not yet completely sunk in soil but it is not usable because it is open (not covered) and children throw everything in it and even small fishes. Today, the well has big fishes inside. The population thought that the well can still be refurbished, normally constructed to be used. However, no NGO or government structure took the initiative to reconstruct this well. Ten years ago, one well was constructed at a distance of 3 kilometers from the village and is a little bit high situated (on a small hill). This well is only one that is used by community members during non-cyclone periods to fetch water for drinking and cooking food in combination with water from the river.

Narrative Summary 6

The organisation 3 through its local committee came to village B to work with local community members to minimize the risk of floods during cyclone hit. They are usually accompanied by the NGO 2 to train local community members in the preparation process and activities contributing to risk reduction. This process of Cyclone Early Warning System (CEWS) was established in 2004 after the passage of cyclone GAFILO that made a lot of damage in this village and that has kept people's memory until today. The Consortium organisation 3 - NGO 2 discussed with local people about their needs to solve the problems. In addition to the training on preparedness to save lives, the consortium worked together with community members to plant trees and Napier grass alongside the river bank to reduce the quantity of flooding water and to protect against erosion that led destruction of houses located near the river, more precisely in 2001. They provided the needed materials for these activities. *"They gave us the necessary materials for planting such as basins, shovels, bags, knives, ropes, etc. [F5-06-M, village B]"*. Unfortunately, some people still keep their houses near the river, and it has been difficult to convince them to move from there. Because of erosion, the river has created another pathway that is very closer to the village. Since 2004, the Consortium has been concentrating efforts on the CEWS communication to save people's lives during cyclone hit. In addition, they started the risk reduction activities since 2021 when they noticed that people were not willing to relocate from their village to another place regardless the severity of floods they were experiencing at every episode of cyclone. *"If only the water level is still at hip height, it is not yet considered serious because it cannot*

enter the house yet. All the inhabitants of this village have already prepared for the floods, and they are always ready to face them because they have decided to stay in the village, and they no longer plan to leave their village and go and live elsewhere [F5-10-F, village B]". Apart of trees and Napier grass, NGO 2 provided sandbags to population that they placed at the river bank. The population said that they were happy with protection that sandbags provided to them by reducing the quantity of flood water. There has been a certain level of improvement since planting trees and Napier grass started. *"Erosion has become a serious problem in our village because there are many houses that are at risk of collapsing, unfortunately we have not been able to convince the people who live very close to the river to abandon their habitats which are at risk of collapsing at any time. In 2001, for example, there were a few houses that collapsed due to erosion. In addition, due to erosion, the river no longer follows its course, but it has now branched off and created a new path that is getting closer and closer to our village. But now the case of erosion decreases a little after plants have been planted on the banks of the river [F5-08-M, village B]"*. The flooding water going to the village come from two sources: Sambirano river and the sea to which the village has access. The Consortium also trained the local population on how to rescue people in case of drowning or fire and how to mount a tent in case of displacement due to a cyclone or other disaster.

<u>Village C</u>

Narrative Summary 7

In village C, at every cyclone event, there is always flooding for which the severity varies according to the season. There are times when the situation is very serious and when it is moderated. The cyclone GAFILO was the deadly and destructive climate event that people won't forget. It have caused deaths and serious damage in the village. Since that moment, people started preparing themselves to cyclone with the support from organisation 3 and NGO 2 providing the relevant information and training for preparedness. When flooding was very severe, water reached the houses' roofs and people were obliged to stay on the roofs for a period of 2 days. The village was flooded for 24 hours but on the second day, the water was not yet completely out of village going back to the river. It was not yet possible for people to leave the roofs to enter inside the houses. During these 2 days, it was not possible to cook food and all the food stocks prepared during the preparedness phases were spoiled by water. When flooding water height was still high, people who live in the surrounding mountains voluntarily brought foods with their traditional wooden boats to those who were blocked in the village by the floods. Most of times, they brought green bananas and animals found died in the flooding water. *"People who lived in the mountains came in wooden boats and fed us with green bananas mixed with meat from the cows that they found drowned during the flood. This food caused stomach aches [F3-06-M, village C]"*. In addition, the catholic church of Bemaneviky organised food delivery to those stuck on their roofs during that difficult periods. *"We ate because we had rice in our depot that had been flooded in water for two days, so only this spoiled rice could be used as food. In order not to starve, we ate spoiled rice while waiting for help. And then the parish priest of the Catholic Church of Bemaneviky arrived to bring us food aid. He gave us maize kernels for each household [F3-03-M, village C]"*. When it was possible to leave the roofs, people did not have food to eat, and they cooked spoiled rice that spent 48 hours flooded in water. Most people had stomachache and diarrhoea after this spoiled food, but they did not have any other choice as that was the only available food they had at that moment. Even if people were living in difficult conditions, staying on houses' roofs for 2 days, they did not take the boats for evacuation with people who brought foods to them. They preferred to stay in the village because they thought that leaving the village would give opportunity to other people to steel their properties.

Narrative Summary 8

After a certain number of floods episodes caused by cyclones in village C, NGO 2 came to discuss with community members of the village to request for places to construct wells and public tap water facilities. After cyclone hit, people did not get support from NGOs in the difficult moment except NGO 2 who refurbished the public primary school and constructed water facilities in the village. Places were given by volunteers who wanted to contribute to have clean water in the village. The challenge is that NGO 2 did everything working with their own staff without involving local people. Community members did not have their voice in the way these facilities should be constructed. During flooding events, these facilities are not usable because they are covered by the flooding water and people are still obliged to use flooding water for their daily life (drinking and cooking food). In addition, NGO 2 has created a Local Rescue Team (Equipe Locale de Secours) and supported them to sensitise community members about cyclone hit. NGO 2 has trained the ELS team in resuscitation in case of drowning before evacuation to hospital. They provided to the team whistles, megaphones, coloured flags, and training. Then the ELS sensitise community members about cyclone hit and what instructions to observe. This process started in 2022 and people have confirmed that since they started receiving these information, there has not been any death during cyclone hit. *"The NGO 2 helped us to prepare well for the arrival of the cyclone. It provided the ELS with equipment such as whistles, megaphones, life jackets, and cyclone flags. And ELS members go around the village informing everyone of the day and time when the cyclone may arrive so that everyone is well prepared. Then some people who live in low places move to a higher place to be safe from the flood [F3-03-F, village C]"*.

Village D

Narrative Summary 9

Inhabitants of village D did not have challenges of drinking water because they have access to wells that were constructed by the NGO 2 which has upgraded three of them to well-pump to be functional very soon. The NGO 2 collaborated with the community for this project by distributing the maintenance task to the local community members. Every person collecting water pays 100 Ariary per day to the volunteer (local community member) who live near the water point and is in-charge of the maintenance process. *"According to what they said, you have to pay 100 Ariary per day per person to the well keeper for maintenance in case of destruction [F4-01-M, village D]"*. People have adhered to this initiative even though some of them are still reluctant to pay that money. This was because people did not expect the repair to be done by the government, that is why they were willing to contribute to solve the problem at their level. *"I don't find any problem because we were not waiting for help from the government to repair damaged things, but among many people there are always those who run away from their responsibilities [F4-01-M, village D]". "There is one person who volunteered, the one who lives near the well. He will collect money from everyone who will take water [F4-01-M, village D]"*.

Narrative Summary 10

After the recent cyclone hit, community members in village D experienced serious difficulties in getting food and other necessities and were not assisted. They have the feeling that no one cared about them thinking that the local authorities did not play their role of reporting the situation of the village allowing the government to be aware of their needs and organise the aid relief. *"When I asked the president of the municipal council about it, he told me that there had never been such a report about the problems we have in our village. So how will the government know the problems we face? How can the government assume its responsibilities or help us solve our problems? As far as I know, no*

one cares about our problems [F4-05-F, village D]". They found that inhabitants of another village in another municipality were supported with food and other important necessities that they could be entitled to because they were in the same situation in term of damage. *"… we did not get even a little food aid while some people in other districts got them after a cyclone hit. For example, the population of the district of AMBILOBE received food and material aid after the passage of the recent cyclone, but we did not get anything while we were also in great difficulty [F4-05-F, village D]"*.

Narrative Summary 11

Community members in village D were seriously affected by cyclone hit through the crops destruction made by repeated floods. They have found this most serious problem for their village and have asked for protection of their crop fields by constructing a defence wall to block water getting into their rice fields. In the past, defence walls were constructed and they have given good results protecting crops that allowed them to produce sufficient quantities of rice in the village for more than 10 years. These defence walls were constructed in 2000 by the government and some by the NGO 5 in 2015 which lasted until 2020, but now, they are destroyed. The villagers have requested many times to officials and MP who visited the village to restore the defence wall as their primary need, but they have not yet got response to their requests. As the MP of the district promised to construct the wall and did not respond, they felt that their request was neglected. *"We made a verbal (but not written) request, and we take advantage of it when there is a candidate who has come to make propaganda especially the MP. He has promised to build the defence when he is elected but so far it has not been done. In my opinion, they don't take our request seriously [F4-01-M, village D]"*. *"Every time a government official passes through here, we always ask for this help to build deference walls. But so far, our requests have not yet been answered. If our rice fields are well restored, our food problems can be significantly reduced [F4-08-M, village D]"*. The villagers estimate that only 30% of rice fields are still cultivable for production while the remaining space is destroyed by floods. During the normal time with wall protection, they were producing 4 tons of rice per ha but now they are producing 1 ton per ha.

Narrative Summary 12

Community members are trying to repair the defence wall and have a local committee with a local leader to supervise the work. However, it has been difficult for them to make progress as they only do it manually with local means using shovels and mud. Hence, they need to get support from NGOs and stakeholders providing sustainable materials such as cement. Community members have verbally asked NGOs intervening in the region to support them and they were expecting their village chief to make a formal request of cement, iron, … but they do not know why this was not done and why they did not answer to their request. They have identified specific spaces of low level in village D where the sea make intrusion in their rice field with different measures: 500m, 200m, 50m, … and in total the spaces make a distance of few kilometers where they try to construct walls. *"The total length of the wall to be built is about 6km, but we will not be able to do it on our own alone. We will never be able to build everything, so we have decided to build walls only in the places where the sea has the most access to the rice fields [F4-04-M, village D]"*. *"We have asked the government but there is no answer, but we have not yet asked NGOs, which is why we are considering asking the NGOs 1 and 2 to help us and provide financial support for the construction of these walls [I-04-M, village D]"*. As the sea level has risen a lot and abundantly, their efforts go in vain as the walls are destroyed by water repeatedly. The committee sensitised all community members to attend the construction work but only a few men attended, and women were not able to do most of the works.

Narrative Summary 13

Community members have the feeling that their local authorities are not supporting their local initiatives that help to solve their specific livelihood problems. There are many young people in village D who have completed their university education and are not employed working as fishers while they could serve at the municipality level. An example was given of a young man who presented his project to the mayor for rice field irrigation that could solve the issue rice field flooding in the village. However, the mayor office did not pay attention to the project, and no response was given. *"… in our village, there are many young people who have baccalaureate, bachelor's or even master's degrees and what is their job now? They are all fishermen. These young people have many good ideas to develop our municipality. For example, the son of participant number 6 had a good project for our rice fields. The rice fields to be restored have already been geolocated on Google Map. However, when this project was announced to the Mayor, he had no reaction. There was no request or support from the mayor to carry out this project, so nothing was done [F4-04-M, village D]"*. Community members have requested both NGOs and government to support them by creating diverse activities on top of the fishing activities to allow diversifying their food and provide jobs to young people.

Narrative Summary 14

Community members in village D were approached by the NGO 1 to organise themselves in a local group for saving to help them managing the difficult periods of cyclone. The group is called GEC (Groupe d'Epargne Communautaire) allowing members to contributing money monthly for saving so that they can borrow when they are in need. This helped them to buy food when their rice fields are destroyed by floods and allowed them to cope with the preparedness process in term of food stock. They may be able to reimburse later when they harvest from their agricultural activities during the non-cyclone periods. This NGO 1 supported them with training to increase their capacity in term of finance management and leadership. It supported them also in raising awareness at the village level to convince community members to join the group and plan for their future. Local people have confirmed that this initiative helped them a lot even though the stock is usually not sufficient to cover the whole period of cyclone-related crisis. *"The GEC initiative has been a great support for us because when our fields of rice are destroyed by floods, we don't have anything to eat and the money we borough there helped us to gather some food during the difficult moment of cyclone, then we pay back later when we produce rice again [F4-06-M, village D]"*.

Narrative Summary 15

Community members usually receive communication about forecasted cyclones for their preparedness and a range of NGOs including NGOs 1, 2, and 4 come together in an awareness and training programme to help local people to prepare and cope the cyclone passage. These NGOs provided materials used for preparedness and monitoring of cyclone events such as whistles, radios, and coloured flags to provide information about cyclone to the population. Local people have confirmed that this preparedness process has helped them a lot to get prepared and their situation has improved compared to the periods before when the system was not implemented. This helped them to avoid loss and damage as they got complete information of what and how to prepare themselves. *"I don't really remember when it was set up, but I can say that the situation of the population has improved with the implementation of this system because everyone is well prepared for the arrival of a cyclone [F4-01-M, village D]"*. Different organisations have organised training for the local population in diverse topics in village D. NGO 2 trained people in cyclone preparedness and monitoring; NGO 1 trained them in marine resources and fisheries management; NGO 4 trained them on leadership and NGO 6 on importance of vaccine. The local people have confirmed that these training helped them a lot to improve their life by protecting the environment and increase their wealth.

Narrative Summary 16

In 2015, the NGO 5 came to village D and initiated a local association that was open to any citizen living in the village to join. The NGO collaborated with local people to build defence walls along the sea to protect the rice fields. These walls provided protection for a duration of five years until 2020 before they got destroyed by the high tides after each cyclone hit. The organisation also supported community members with fish nets, machine to husk rice, machine to make planks, and engines for boat. The local association that was created, made activities after getting support from the NGO 5 and was able to gain money that was used to solve minor problems of the village. The collaboration with NGO 5 has stopped and the engines are no longer operational.

Narrative Summary 17

Having worked for several years elsewhere in the Commune, the NGO 1 asked the Commune leaders for permission to work in the village D - then came to the village leaders to ask them directly. The NGO 1 came into fishing village D - to tackle mangrove destruction and depletion of fishing stocks. Fish and shrimps breed in the mangroves and mangroves also provide a natural barrier against flood waters. Having made contact, the NGO negotiated with the villagers, asking what they needed and providing a donation of cooking materials such as plates, pots, ladles, spoons, bowls in return for community members joining in to replant 5 hectares of mangroves. Respondents described how "*The community is very dynamic in the execution of the activities carried out by the NGO 1 due to the motivations it brings to the community.*" The key informant (a local government representative from the village) hinted that they could have negotiated more but "*We didn't ask for great things for fear that we wouldn't get them.*"

Narrative Summary 18

Working with community members, the NGO 1 provided training on marine resource management and established CLB (Local Base Committee) which is responsible for managing and monitoring the mangrove forest and controlling the types of nets that fishermen use. This has contributed to protection of the mangrove forests which respondents reported that it is *almost restored* and "due to *this restoration, we have noticed that there is an increase in our production [of fish, crabs and shrimp*]". The NGO 1 also encouraged local ownership and decision making, over time: "*First of all, the NGO 1 convinced us to restore our mangrove forest and it also helped us with the protection of these mangroves. Then, they also encouraged us to respect the closure period. Due to the awareness-raising they have done, we have decided with the collaboration of the neighbouring village of AN- the closing dates and the opening date of fishing in our fishing area. […] we and the villagers of AN- decided on the dates together*."

Narrative Summary 19

Protection of mangroves and the banning of fishing during breeding times (March-May) have resulted in better sustained stocks and therefore more income leading to greater food security:
"After the closure we have more production and we can sell more fish, so we can earn a lot more money to improve our lives. During the opening we can buy equipment such as brand new fishing nets, even buy canoes, buy food, clothes and furniture for our families. We can also save money to buy food during the rainy season."

People say they trust the NGO 1 because they *"have helped us a lot and have accompanied us for a long time in our difficulties."*

Nevertheless, challenges remain. Illegal felling of wood from the mangroves is still happening as is illegal fishing by people from other villages. These actions continue to affect fishing stocks and there are tensions between the two villages D and AN regarding the use of nets with smaller holes (which catch small fish, depleting the stocks) with people looking to the government to enforce these locally set restrictions. The representative of village D says "*I talk by phone to the village chief of AN- but he said that the villagers don't want to follow the rules […] if a member of the government arrives, I think they will give in*". More pragmatically, FGD respondents wanted the NGO to provide a speedboat so that they can patrol the protected forests and the KI noted the need for a paid "guardian" to monitor this.

Village E

Narrative Summary 20

Since 2004, After cyclone GAFILO hit, the village E was seriously damaged and all the following cyclones in this village are usually accompanied by floods. The village is located closer to both the sea and the river Sambirano. Flooding water come from these two sources. The flooding destroyed both houses and livelihoods putting people in difficult situations. They could not find easily food to eat during cyclone periods because all the fields of rice, banana, and casava were flooded. The village has no clean water points and travel to a distance of three kilometers to fetch water. When the village is flooded, people stay in their houses and use flooding water which is always contaminated. *"What I want to add to what has already been said, we have had water problems. We drank the water of the Sambirano river which overflowed and flooded our village. We had no other option than drink the dirty water with died animals and the dirty. There was no place where we could look for clean water. We drank really dirty water [F2-04-M, village E]"*. During each cyclone hit, people became sick developing diarrhoea, respiratory diseases, and fever. Most people in the village do not boil water before use. Domestic animals are also killed by floods. The floods are becoming more severe at each cyclone hit and people construct their houses with long pillars of 1.5 – 2 meters to allow them staying in their houses even when the village is flooded. People do not want to leave the village because they have all their investments there: farm, domestic animals, that they try to protect for their future life. As the village is closer to the sea, there are frequent minor to moderate floods that occur two to three times per week in the village due high tides. People are used to this situation, and they take precautions to stay in their houses during that time. These frequent floods last 2 to 3 hours then water goes back to sea. *"When they build houses, they make houses with long feet to bring the houses high up to avoid water. And they are used to water that comes and goes. They're already used to it because it's their life. They fish, that's their life here. The water that floods the village arrives very early morning in the village and it lasts 2 to 3 hours before going back to the sea [K-04-F, village E]"*.

Narrative Summary 21

The NGO 2 has supported community members to clean the well that they use to fetch water and distributed product for water treatment (sur'eau). During that time, cases of diarrhoea decreased. Now, it has been 4 years without cleaning the well and diarrhoea cases are increasing again. The well cleaning was done since 2014 until 2020, and it has been 4 years without cleaning because the person in-charge did not mobilise community members for this activity. Local people misbehaviour urinating and poo around the well and clean babies cloths containing poo near the well. Before, there was a fence around the well , which is now destroyed. At that moment, people had clear schedule of fetching water to avoid these misbehaviours. Only a few households have latrines in the villages, and most people use their immediate environment for poo and urine. *"… they do many things such as*

bathe and urinate near the well, wash clothes soiled with baby feces in the well yard. In addition, here there are not many latrines and some people during the night would go to urinate and defecate there, not far from the well [F2-03-F, village E]".

Narrative Summary 22

The NGO 2 has trained local people to become mobilisers for cyclone preparedness and during cyclone hit. These people go around the village sensitizing community members on what to prepare before cyclone passage. They provide materials such whistles, life-vests, coloured cyclone flags, megaphones, and mobile phones to alert people about cyclone passage but also the flooding water from the sea or the river. When these mobilisers get information by phone, they raise the appropriate flag and alert community members. However, people thought that these materials are not sufficient because there are moments when flooding water arrives suddenly in night when people are sleeping and at that time, mobilisers have to go door-to-door to wake up everyone in the village. They need to have a boat that they can use to reach many people easily and without delay. This boat will allow them also to go far to sensitise people who live in the forest which is flooded very quickly. People who live there refuse to leave their houses and they want to take care of their animals and lands. *"The mobilizers only shouted and used whistles during the awareness-raising session. There are times when the water arrives unexpectedly during the night, and some people are already sleeping. And the mobilizers will sometimes go door to door to wake up and alert the inhabitants. But they do not have means, such as canoes or speedboats, to alert people who live a little far from the village [F2-01-M, village E]"*. The following instructions are given to community members for cyclone preparedness: everyone must be sure that s/he is located in a safe place or move to a designated place, every household must prepare sufficient stock of food, move all the domestic animals to the mountains, follow the information on radio about the progress of cyclone, and tie the wooden boat to the houses. The NGO 2 provided small radio to households which did not have them allowing them to follow cyclone progress.

All villages

Narrative Summary 23

Under the cyclone surveillance system, when there is information about cyclone which is about to hit a region, the organisation 3 communicate directly to the local authorities at the district level with all the details about the path, speed, duration. Then, the district authorities communicate to the local authorities at the villages concerned and NGOs that support them. These local authorities and the NGOs gather and communicate with the community members using whistles, megaphones, and coloured flags (4 colours). This help them to closely monitor the cyclone event at different steps. The green flag is raised to show that the cyclone is about to pass in the village in a period of 14 to 10 days. During this period, stakeholders (NGOs such as NGOs 2, 3 and 7, and local authorities) organise community dialogue with locals to advise them to prepare sufficient stock of food and water that can last for at least one month. Advice is given to locals by NGO 2 and the local committee of organisation 3 to protect all the valuable documents/ products such as education degree, property documents, money by putting them in a plastic waterproof bag and placing them at a safe place where water cannot reach. Community members start reinforcing their houses' roofs at this step and engage in risk reduction activities. The yellow flag is raised to show that the cyclone will reach the village very soon in the next 3 to 2 days. At this step all the activities are closed, including schools, market, farming and fishing activities. Everyone must stay at home and follow closely information on radio and the flag colour changes. People who live in fragile houses or in unsafe places must move to either neighboring fostering houses or to a chosen safe place such a school which is placed on a high space such as a

mountain. The red flag is raised to mean that the cyclone is in the village, and every household must ensure that the family members are all in the house at a safe place with doors and windows closed. People continue listening to the radio to get real time communication about risk. Then the blue flag is raised to explain that the cyclone is over, and people can now go out of the houses but continue to follow closely the communication on radio as the cyclone can still come back and there must be post-cyclone crises or damages that need people to be aware of such as floods, landslides, road and/or health facility destruction.
