# Supplementary material for: The role of embedded Non-Governmental Organisations and other stakeholders in building resilience to cyclone-related crises in Madagascar: a qualitative study
Source: BMC Glob Public Health. 2026 Jul 9;4:66. doi: 10.1186/s44263-026-00300-y (PMC13348428; doi:10.1186/s44263-026-00300-y)
Supplement: Supplementary file 4 — Supplementary Material 4: COREQ Checklist. [file 44263_2026_300_MOESM4_ESM.pdf]

Topic Guide for FGD, English Version

Study title: Community Engagement, Resilience, and Partnership for Health Responses in Cyclone-Affected Settings: Evidence from Madagascar

Principal Investigator: Dr. Mateus Kambale Sahani
Supervisors: Dr Neha Singh, Prof Susannah Mayhew, and Prof Janet Seeley

Topic guides FGD (for community members)

I. Identification

ID number: ………………… **Age:** …………. (years) **Sex:** Male/ Female/ Others (specify):……………………
Level of education: reached primary/ reached secondary school/ reached the university

Residency (Commune, Village):
1. Commune Ambalahonko (village: Ambiky) 2. Commune Ankatafa (village: Ankatafabe) 3. Commune Bemanevika Haute Sambirano (village: Ambodifinesy) 4. Commune Maherivaratra (village: Ampampamena) 5. Commune Ambohimena (village: Ampahakabe) 6. Others (specify): …………………

Social status: 1. Teacher 2. Business person 3. Farmer 4. Student 5. Health professional 6. Fisher 7. Others (specify): …………………………………………………………

II. Questions
Have you experienced any health crisis such as cyclone, epidemic (apart of COVID-19), drought or flood in this community? If yes, please describe how it affected you and what you did to cope with it.
Probes only if needed but encourage all participants to contribute their views on the questions above
- How did it affect your community? (e.g. food/water shortages; destruction of roads etc.)
- How did you cope with this situation?
- Were there any actions – by you or others – put in place to solve the issue? What were these actions and what was their purpose?
- In your view, were these actions successful?
- If yes, what made them successful?

When [crisis mentioned in previous question] was affecting you, were any of you personally involved in the actions you described [i.e. in the previous question]? If so, please describe what you did and how and why you came to be involved.

Let people talk about their involvement then use the probes only if necessary:

- Were you invited?
  - If yes, who invited you? In what circumstances? What was your role? Did you have any involvement in planning or decision making e.g. about what actions to take?
  - If no, did you start a response (actions) yourself? Did you ask anyone else for help?
- What stimulated you to be engaged in the response?
- Were your contributions considered by the people who invited you to participate?
  - If yes, how did they help to solve the issue?

- If no, why were they not taken into account?

Are there any of you who were not personally involved in any of these actions? [*or if there aren't then ask whether there were any other community members who were not involved*]. If so (never participated), would you be willing to take part in activities responding to future health/environment crisis (epidemic, drought, flooding, cyclone, etc.)?

- What would motivate you to participate?
- What sort of activities would you be interested in being involved with?
- Would you wait to be invited to participate? If yes, who should invite you to make you comfortable to participate?

Do you get communication from official/meteorological authorities about cyclones hitting for your preparedness? If yes, is it done well in advance and how do you prepare yourself? Do you get support from NGO or government for your preparedness? What kind of support?

Do you get communication from official/meteorological authorities about projection of famine crisis for your preparedness? If yes, is it done well in advance and how do you prepare yourself? Do you get support from NGO or government for your preparedness? What kind of support?

Who do you trust to help you respond to these challenges you face? Please explain more.
How easy is it for people in this community to initiate actions to solve a crisis before getting any support from the government and/or NGO?

- If yes, have you done this and what did you do?
- If No, why is it not possible?

Have you (or others in this community) ever worked with government people or NGO/health staff to respond to health/environment crisis (epidemic, drought, flooding, cyclone, etc.) in this community?

- If yes, please give details (who did you collaborate with, how and why? Who initiated the collaboration? What was the nature of the collaboration)
- Is there anything you would need or want from government or NGO for you to get ready any time to intervene when there is a health or climate-related crisis in this community?
- Have you ever been invited for a community dialogue for a crisis response? If yes, please give details (who invited you, for what purpose, what was the result of the dialogue?)
- Have you or any other members of your community ever been trained by government/NGO people to respond to health/climate related crisis? If yes, please give details (who trained you, what did they train in and what was the purpose; what are your views on the training offered?)
- Have NGO or government people ever recruited any of your community members to implement activities to respond to a health/climate related emergency? If yes, please give details (who recruited people and for what purpose; which people were recruited and how).

Did any of the crises impact you economically?

- What happened?
- What did you do to rebuild your economy?

- Did you get support? If yes, from whom?

Are you aware of the existing guidelines for the construction of wooden houses allowing resistance to cyclones or storm? If yes, how did you know about them? Are there people or NGO who speak to you about them? How did you come in interaction with them?

How do you find the fishing areas closure or protected areas policies in your community? Do they help you in developing your activities and increase your production? How are they implemented? Are you involved in the implementation? Are the protected areas or the closure dates determined jointly with you? Do you think the policies could be implemented differently?

Is there a group of people in your community ready any time to respond to a health/climate related crisis?

- If yes, who are they, how were they established and how do they work?
- Do they have permanent activities relating to preparing for and responding to crises?
- What is being done to maintain them active and useful?
- Are they in contact with (or do they receive any support from) donors and/or government?
- If they are not there, are they needed? Why?

Have you – or others in this community – ever had contact with or worked with Blue Ventures or any of their mobilisers? If yes, please describe how you came to engage with them and what you have done with them. *They may have talked about Blue Ventures in response to the Qn on NGO contact, in which case check the probes and ask further details if necessary. If they have not yet mentioned Blue Ventures then let them talk first, but then probe for details on the following if it has not been mentioned:*

- Blue Ventures staff directly approached you?
- Attended Blue Ventures events or services?
- Attended community level events or other events organised by BV or other NGO?

In your interactions with Blue Ventures, were you able to tell them what you wanted or needed? Were you ever part of the planning or decision making about actions being undertaken? Please give as much detail as possible.

Overall, what is your view of how helpful Blue Ventures (or other NGO) activities are in helping to support your community to respond to the challenges you have faced during cyclones or epidemics?

- Are there any positive aspects of how Blue Ventures or other NGOs have supported crisis-response in this community? (e.g. did they listen to what community wanted? Did they involve them in planning/decision making?)
- What could these NGOs do better/differently (including how they interact with communities and the extent to which they include them in decision making and developing plans for preparing to respond to any crises)

Is there anything else you would like to say about responding to the health or climate crises that you face in this community?   Thank you for your time

Topic Guide FGD, French version

Titre de l'étude : Engagement communautaire, résilience et partenariat pour les réponses sanitaires dans les contextes touchés par les crises de cyclones : études de cas de Madagascar

Chercheur principal : Dr Mateus Kambale Sahani
Superviseurs : Dr Neha Singh, Prof Susannah Mayhew, and Prof Janet Seeley

Guides thématiques FGD (pour les membres de la communauté)

I. Identification

Numéro d'identification : .... **Âge :** .... (années) **Sexe :** M/F/Autres (préciser) :.................

Niveau d'éducation : a atteint l'école primaire / a atteint l'école secondaire / a atteint l'université

Résidence (Commune, Village) :

1. Commune Ambalahonko (village: Ambiky) 2. Commune Ankatafa (village: Ankatafabe) 3. Commune Bemanevika Haute Sambirano (village: Ambodifinesy) 4. Commune Maherivaratra (village: Ampampamena) 5. Commune Ambohimena (village: Ampahakabe) 6. Others (specify): …………………

Statut social : 1. Enseignant 2. Homme d'affaires 3. Agriculteur 4. Étudiant 5. Professionnel de la santé 6. Pêcheur 7. Autres (préciser) : ....

II. Questions

Avez-vous vécu une crise sanitaire telle qu'un cyclone, une épidémie (à l'exception de COVID-19), une sécheresse ou une inondation dans cette communauté ? Si oui, veuillez décrire comment cela vous a affecté et ce que vous avez fait pour y faire face.
Ne sondez que si nécessaire, mais encouragez tous les participants à donner leur point de vue sur les questions ci-dessus

- Comment cela a-t-il affecté votre communauté ? (par exemple, pénuries de nourriture et d'eau, destruction de routes, etc.)
- Comment avez-vous fait face à cette situation ?
- Des actions – de votre part ou de celles d'autres personnes – ont-elles été mises en place pour résoudre le problème ? Quelles étaient ces actions et quel était leur but ?
- À votre avis, ces actions ont-elles été couronnées de succès ?
- Si oui, qu'est-ce qui a fait leur succès ?

Lorsque [la crise mentionnée dans la question précédente] vous a affecté, l'un d'entre vous a-t-il été personnellement impliqué dans les actions que vous avez décrites [c'est-à-dire dans la question précédente] ? Si oui, veuillez décrire ce que vous avez fait et comment et pourquoi vous en êtes venu à vous impliquer.

Laissez les gens parler de leur implication puis n'utilisez les sondes que si nécessaire :

- Avez-vous été invité ?

    - Si oui, qui vous a invité ? Dans quelles circonstances ? Quel était votre rôle ? Avez-vous participé à la planification ou à la prise de décision, par exemple sur les mesures à prendre ?
    - Si non, avez-vous commencé une réponse (actions) vous-même ? Avez-vous demandé de l'aide à quelqu'un d'autre ?
- Qu'est-ce qui vous a incité à vous engager dans la réponse ?
- Vos contributions ont-elles été prises en compte par les personnes qui vous ont invité à participer ?
    - Si oui, comment ont-ils aidé à résoudre le problème ?
    - Si non, pourquoi n'ont-ils pas été pris en compte ?

Y a-t-il des personnes qui n'ont pas été personnellement impliquées dans l'une de ces actions ? [*ou s'il n'y en a pas, demandez s'il y avait d'autres membres de la communauté qui n'étaient pas* impliqués]. Si oui (n'avez jamais participé), seriez-vous prêt à participer à des activités répondant à une future crise sanitaire/environnementale (épidémie, sécheresse, inondation, cyclone, etc.) ?

- Qu'est-ce qui vous motiverait à participer ?
- Dans quel genre d'activités aimeriez-vous participer ?
- Attendriez-vous d'être invité à participer ? Si oui, qui devrait vous inviter pour vous mettre à l'aise pour participer ?

Recevez-vous des communications des autorités officielles/météorologiques sur les cyclones qui frappent pour votre préparation ? Si oui, est-ce fait bien à l'avance et comment vous préparez-vous ? Recevez-vous le soutien d'ONG ou du gouvernement pour votre préparation ? Quel type de soutien ?

Recevez-vous des communications des autorités officielles/météorologiques sur la projection d'une crise de famine pour votre préparation ? Si oui, est-ce fait bien à l'avance et comment vous préparez-vous ? Recevez-vous le soutien d'ONG ou du gouvernement pour votre préparation ? Quel type de soutien ?

À qui faites-vous confiance pour vous aider à relever ces défis auxquels vous êtes confrontés ? Expliquez-nous davantage.

Est-il facile pour les membres de cette communauté d'initier des actions pour résoudre une crise avant d'obtenir le soutien du gouvernement et/ou de l'ONG ?

- Si oui, l'avez-vous fait et qu'avez-vous fait ?
- Si non, pourquoi n'est-ce pas possible ?

Avez-vous déjà travaillé avec des membres du gouvernement ou des ONG/personnel de santé pour répondre à une crise sanitaire/environnementale (épidémie, sécheresse, inondation, cyclone, etc.) dans cette communauté ?

- Si oui, veuillez donner des détails (avec qui avez-vous collaboré, comment et pourquoi ? Qui a initié la collaboration ? Quelle était la nature de la collaboration)
- Y a-t-il quelque chose dont vous auriez besoin ou que vous voudriez de la part du gouvernement ou d'une ONG pour que vous soyez prêt à intervenir à tout moment en cas de crise sanitaire ou climatique dans cette communauté ?

- Avez-vous déjà été invité à un dialogue communautaire pour une réponse à une crise ? Si oui, veuillez donner des détails (qui vous a invité, dans quel but, quel a été le résultat du dialogue ?)
- Avez-vous déjà été formé(e) par des représentants du gouvernement/d'ONG pour répondre à une crise liée à la santé ou au climat ? Si oui, veuillez donner des détails (qui vous a formé, en quoi ont-ils été formés et quel était le but ; quel est votre point de vue sur la formation proposée ?)
- Des ONG ou des représentants du gouvernement ont-ils déjà recruté des membres de votre communauté pour mettre en œuvre des activités visant à répondre à une urgence sanitaire/climatique ? Dans l'affirmative, veuillez donner des détails (qui a recruté des personnes et dans quel but ; quelles personnes ont été recrutées et comment).

L'une des crises vous a-t-elle affecté économiquement ?
- Que s’est-il passé?
- Qu'avez-vous fait pour reconstruire votre économie ?
- Avez-vous reçu du soutien ? Si oui, de qui ?

Connaissez-vous les directives existantes pour la construction de maisons en bois permettant de résister aux cyclones ou aux tempêtes ? Si oui, comment les avez-vous connus ? Y a-t-il des personnes ou des ONG qui vous en parlent ? Comment êtes-vous entré en contact avec eux ?

Comment trouvez-vous les zones de pêche fermées ou les politiques sur les aires protégées dans votre communauté ? Vous aident-ils à développer vos activités et à augmenter votre production ? Comment sont-ils mis en œuvre ? Êtes-vous impliqué dans la mise en œuvre ? Les aires protégées ou les dates de fermeture sont-elles déterminées conjointement avec vous ? Pensez-vous que ces politiques pourraient être mises en œuvre différemment ?

Y a-t-il un groupe de personnes, dans votre communauté, prêt à tout moment à répondre à une crise liée à la santé ou au climat ?
- Si oui, qui sont-ils, comment ont-ils été créés et comment fonctionnent-ils ?
- Ont-ils des activités permanentes liées à la préparation et à la réponse aux crises ?
- Que fait-on pour les maintenir actifs et utiles ?
- Sont-ils en contact avec les donateurs et/ou le gouvernement (ou reçoivent-ils un soutien de leur part) ?
- S'ils ne sont pas là, sont-ils nécessaires ? Pourquoi?

Avez-vous – ou d'autres membres de cette communauté – déjà eu des contacts ou travaillé avec Blue Ventures ou l'un de leurs mobilisateurs ? Si oui, veuillez décrire comment vous en êtes venu à vous engager avec eux et ce que vous en avez fait. *Ils ont peut-être parlé de Blue Ventures en réponse au Qn sur le contact avec les ONG, auquel cas vérifier les enquêtes et demander plus de détails si nécessaire. S'ils n'ont pas encore mentionné Blue Ventures, laissez-les parler d'abord, mais ensuite demandez des détails sur les points suivants si cela n'a pas été mentionné :*

- Le personnel de Blue Ventures vous a directement approché ?
- Vous avez assisté à des événements ou à des services de Blue Ventures ?
- Vous avez participé à des événements communautaires ou à d'autres événements organisés par BV ou une autre ONG ?

Dans vos interactions avec Blue Ventures, avez-vous pu leur dire ce que vous vouliez ou ce dont vous aviez besoin ? Avez-vous déjà participé à la planification ou à la prise de décision concernant les actions entreprises ? Veuillez donner autant de détails que possible.

Dans l'ensemble, que pensez-vous de l'utilité des activités de Blue Ventures (ou d'autres ONG) pour aider votre communauté à répondre aux défis auxquels vous avez été confronté lors de cyclones ou d'épidémies ?

- Y a-t-il des aspects positifs dans la façon dont Blue Ventures ou d'autres ONG ont soutenu la réponse aux crises dans cette communauté ? (par exemple, ont-ils écouté ce que la communauté voulait ? Les ont-ils impliqués dans la planification/prise de décision ?)
- Que pourraient faire ces ONG mieux ou différemment (y compris la façon dont elles interagissent avec les communautés et la mesure dans laquelle elles les incluent dans la prise de décision et l'élaboration de plans pour se préparer à répondre à toute crise)

Y a-t-il autre chose que vous aimeriez dire sur la réponse aux crises sanitaires ou climatiques auxquelles vous êtes confrontés dans cette communauté ?

Merci pour votre temps.

Topic Guide IDI, English version

Study title: Community Engagement, Resilience, and Partnership for Health Responses in Cyclone-Affected Settings: Evidence from Madagascar

Principal Investigator: Dr. Mateus Kambale Sahani
Supervisors: Dr Neha Singh, Prof Janet Seely, and Prof Susannah Mayhew

Topic guides IDI (for community members)

I. Identification

ID number: ………………… **Age:** …………. (years) **Sex:** Male/ Female/ Others (specify):………………………..

Level of education: reached primary/ reached secondary school/ reached the university

Residency (Commune, Village):

1. Commune Ambalahonko (village: Ambiky) 2. Commune Ankatafa (village: Ankatafabe) 3. Commune Bemanevika Haute Sambirano (village: Ambodifinesy) 4. Commune Maherivaratra (village: Ampampamena) 5. Commune Ambohimena (village: Ampahakabe) 6. Others (specify): …………………

Social status: 1. Teacher 2. Business person 3. Farmer 4. Student 5. Health professional 6. Fisher 7. Others (specify): ……………………………………………………

II. Questions

Have you experienced any health crisis such as cyclone, epidemic (apart of COVID-19), drought or flood in this community? If yes,

- How did it affect your community? (e.g. food/water shortages; destruction of roads etc.)
- How did you cope with this situation?
- Were any actions – by you or others – put in place to solve the issue? What were these actions and what was their purpose?
- In your view, were these actions successful?
- If yes, what made them successful?

Have you ever taken part in activities responding to health/environment crisis (epidemic, drought, flooding or their consequences like food (fish or others)/water shortage or roads destruction)?

If yes (participated), please describe what you did and how you came to be involved.

- Were you invited?
    - If yes, Who invited you? In what circumstances? What was your role? Did you have any involvement in planning or decision making e.g. about what actions to take?
    - If no, did you start a response/actions yourself? Did you ask anyone else for help?
- What stimulated you to be engaged in the response?
- Were your contributions considered by the people who invited you to participate?
    - If yes, how did they help to solve the issue?
    - If no, why were they not taken into account?

If no (never participated), would you be willing to take part in activities responding to health/environment crisis (epidemic, drought, flooding, storm, etc.)?

- What would motivate you to participate?
- What sort of activities would you be interested in being involved with?
- Would you wait to be invited to participate? If yes, who should invite you to make you comfortable to participate?

Are there those that you trust the most and those that you do not trust to help you respond to these challenges you face? Please explain more.

Do you get communication from official/meteorological authorities about cyclones hitting for your preparedness? If yes, is it done well in advance and how do you prepare yourself? Do you get support from NGO or government for your preparedness? What kind of support?

Do you get communication from official/meteorological authorities about projection of famine crisis for your preparedness? If yes, is it done well in advance and how do you prepare yourself? Do you get support from NGO or government for your preparedness? What kind of support?

Is it possible for you to initiate actions to solve a crisis on your own or with other members of the community before getting any support from the government and/or NGO?

- If yes, have you done this and what did you do?
- If No, why is it not possible?

Have you ever worked with government people or NGO/health staff to respond to health/environment crisis (epidemic, drought, flooding, cyclone, etc.) in this community?

- If yes, please give details (who did you collaborate with, how and why? Who initiated the collaboration? What was the nature of the collaboration)
- Have you ever been invited for a community dialogue for a crisis response? If yes, please give details (who invited you, for what purpose, what was the result of the dialogue?)
- Have you or any other members of your community ever been trained by government/NGO people to respond to health/climate related crisis? If yes, please give details (who trained you, what did they train in and what was the purpose; what are your views on the training offered?)
- Have NGO or government people ever recruited any of your community members to implement activities to respond to a health/climate related emergency? If yes, please give details (who recruited people and for what purpose).

Did any of the crises impact you economically?
- What happened?
- What did you do to rebuild your economy?
- Did you get support? If yes, from whom?

Are you aware of the existing guidelines for the construction of wooden houses allowing resistance to cyclones or storm? If yes, how did you know about them? Are there people or NGO who speak to you about them? How did you come in interaction with them?

How do you find the fishing areas closure or protected areas policies in your community? Do they help you in developing your activities and increase your production? How are they implemented? Are you involved in the implementation? Are the protected areas or the closure dates determined jointly with you? Do you think the policies could be implemented differently?

Is there a group of people in your community ready any time to respond to a health/climate related crisis (floods, landslides, drought, epidemic)?
- If yes, who are they, how were they established and how do they work?
- Do they have permanent activities relating to preparing for and responding to crises?
- What is being done to maintain them active and useful?
- Are they in contact with donors and/or government?
- If they are not there, are they needed? Why?

Have you ever had contact with or worked with Blue Ventures or other NGO or any of their mobilisers? If yes, please describe how you came to engage with them and what you have done with them. *Probe for details on the following if they are not mentioned but also let them add other things*

- Blue Ventures staff directly approached you [*ask what for and how and why the person responded to Blue Ventures]*
- Attended Blue Ventures events or services

In your interactions with Blue Ventures, were you able to tell them what you wanted or needed? Were you ever part of the planning or decision making about actions being undertaken? Please give as much detail as possible.

Overall, what is your view of how helpful Blue Ventures/other NGO activities are helping to support your community to respond to the challenges you have faced?

- Are there any positive aspects of how Blue Ventures/ other NGOs support crisis-response in this community? (e.g. did they listen to what community wanted? Did they involve them in planning/decision making?)
- What could these NGOs do better/differently (including how they interact with communities and the extent to which they include them in decision making and developing plans for preparing to respond to any crises)

What can be done to make actions sustainable for the response to climate/environment crises?

Is there anything else you would like to say about responding to the health or climate crises that you face in this community?

Thank you for your time.

<u>Topic Guide IDI, French version</u>

Titre de l'étude : Engagement communautaire, résilience et partenariat pour les réponses sanitaires dans les contextes touchés par les crises de cyclones : études de cas de Madagascar

Chercheur principal : Dr Mateus Kambale Sahani
Superviseurs : Dr Neha Singh, Prof Susannah Mayhew, and Prof Janet Seeley

Guides thématiques IDI (pour les membres de la communauté)

<u>I. Identification</u>

Numéro d'identification : .... **Âge :** .... (années) **Sexe :** H/F/Autres (préciser): ....

Niveau d'éducation : a atteint l'école primaire / a atteint l'école secondaire / a atteint l'université

Résidence (Commune, Village) :

1. Commune Ambalahonko (village: Ambiky) 2. Commune Ankatafa (village: Ankatafabe) 3. Commune Bemanevika Haute Sambirano (village: Ambodifinesy) 4. Commune Maherivaratra (village: Ampampamena) 5. Commune Ambohimena (village: Ampahakabe) 6. Others (specify): …………………

Statut social : 1. Enseignant 2. Homme d'affaires 3. Agriculteur 4. Étudiant 5. Professionnel de la santé 6. Fisher 7. Autres (préciser) : ....

II. Questions

Avez-vous vécu une crise sanitaire telle qu'un cyclone, une épidémie (à l'exception de COVID-19), une sécheresse ou une inondation dans cette communauté ? Si oui,

- Comment cela a-t-il affecté votre communauté ? (par exemple, pénuries de nourriture et d'eau, destruction de routes, etc.)
- Comment avez-vous fait face à cette situation ?
- Des actions – de votre part ou de celles d'autres personnes – ont-elles été mises en place pour résoudre le problème ? Quelles étaient ces actions et quel était leur but ?
- À votre avis, ces actions ont-elles été couronnées de succès ?
- Si oui, qu'est-ce qui a facilité leur succès ?

Avez-vous déjà participé à des activités répondant à des crises sanitaires/environnementales (épidémie, sécheresse, inondations ou leurs conséquences sur la production de la nourriture (poissons ou autres)/ pénurie d'eau ou destruction de routes) ?

Si oui (a participé), veuillez décrire ce que vous avez fait et comment vous en êtes venu à vous impliquer.

- Avez-vous été invité?
    - Si oui, qui vous a invité ? Dans quelles circonstances ? Quel était votre rôle ? Avez-vous participé à la planification ou à la prise de décision, par exemple sur les mesures à prendre ?
    - Si non, avez-vous commencé des actions vous-même ? Avez-vous demandé de l'aide à quelqu'un d'autre ?
- Qu'est-ce qui vous a incité à vous engager dans la réponse à la crise sanitaire ?
- Vos contributions ont-elles été prises en compte par les personnes qui vous ont invité à participer ?
    - Si oui, comment ont-ils aidé à résoudre le problème ?
    - Si non, pourquoi n'ont-ils pas été pris en compte ?

Si non (n'a jamais participé), seriez-vous prêt à participer à des activités en réponse à une crise sanitaire/environnementale (épidémie, sécheresse, inondation, tempête, etc.) ?

- Qu'est-ce qui vous motiverait à participer ?
- Dans quel genre d'activités aimeriez-vous participer ?
- Attendriez-vous d'être invité pour participer ? Si oui, qui devrait vous inviter pour vous mettre à l'aise à participer ?

Y a-t-il ceux en qui vous avez le plus confiance et ceux en qui vous n'avez pas confiance pour vous aider à répondre à ces défis auxquels vous êtes confrontés ? Expliquez-nous davantage.

Recevez-vous des communications des autorités officielles/météorologiques sur les cyclones qui frappent votre région pour votre préparation ? Si oui, est-ce fait bien à l'avance et comment vous préparez-vous ? Recevez-vous le soutien d'ONG ou du gouvernement pour votre préparation ? Quel type de soutien ?

Recevez-vous des communications des autorités officielles/météorologiques sur la projection d'une crise de famine pour votre préparation ? Si oui, est-ce fait bien à l'avance et comment vous préparez-vous ? Recevez-vous le soutien d'ONG ou du gouvernement pour votre préparation ? Quel type de soutien ?

Est-il possible pour vous d'initier des actions pour résoudre une crise a votre niveau ou avec d'autres membres de la communauté avant d'obtenir le soutien du gouvernement et/ou des ONG ?

- Si oui, l'avez-vous fait et qu'avez-vous fait ?
- Si non, pourquoi n'est-ce pas possible ?

Avez-vous déjà travaillé avec des représentants du gouvernement ou des ONG/personnel de santé pour répondre à une crise sanitaire/environnementale (épidémie, sécheresse, inondation, cyclone, etc.) dans cette communauté ?

- Si oui, veuillez donner des détails (avec qui avez-vous collaboré, comment et pourquoi ? Qui a initié la collaboration ? Quelle était la nature de la collaboration)
- Avez-vous déjà été invité à un dialogue communautaire pour une réponse à une crise ? Si oui, veuillez donner des détails (qui vous a invité, dans quel but, quel a été le résultat du dialogue ?)
- Avez-vous déjà été formé(e) par des représentants du gouvernement/d'ONG pour répondre à une crise liée à la santé ou au climat ? Si oui, veuillez donner des détails (qui vous a formé, en quoi ont-ils été formés et quel était le but ; quel est votre point de vue sur la formation proposée ?)
- Des ONG ou des représentants du gouvernement ont-ils déjà recruté des membres de votre communauté pour mettre en œuvre des activités visant à répondre à une urgence sanitaire/climatique ? Dans l'affirmative, veuillez donner des détails (qui a recruté des personnes et dans quel but).

L'une des crises vous a-t-elle affecté économiquement ?

- Que s’est-il passé?
- Qu'avez-vous fait pour reconstruire votre économie ?
- Avez-vous reçu du soutien ? Si oui, de qui ?

Connaissez-vous les directives existantes pour la construction de maisons en bois permettant de résister aux cyclones ou aux tempêtes ? Si oui, comment les avez-vous connus ? Y a-t-il des personnes ou des ONG qui vous en parlent ? Comment êtes-vous entré en contact avec eux ?

Comment trouvez-vous les zones de pêche fermées ou les politiques sur les aires protégées dans votre communauté ? Vous aident-ils à développer vos activités et à augmenter votre production ? Comment sont-ils mis en œuvre ? Êtes-vous impliqué dans la mise en œuvre ? Les aires protégées ou les dates de fermeture sont-elles déterminées conjointement avec vous ? Pensez-vous que ces politiques pourraient être mises en œuvre différemment ?

Y a-t-il un groupe de personnes dans votre communauté qui est prêt à tout moment à répondre à une crise sanitaire/climatique (inondations, glissements de terrain, sécheresse, épidémie) ?

- Si oui, qui sont-ils, comment ont-ils été créés et comment fonctionnent-ils ?
- Ont-ils des activités permanentes liées à la préparation et à la réponse aux crises ?
- Que fait-on pour les maintenir actifs et utiles ?
- Sont-ils en contact avec les donateurs et/ou le gouvernement ?

- S'ils ne sont pas là, sont-ils nécessaires ? Pourquoi?

Avez-vous déjà eu des contacts ou travaillé avec Blue Ventures ou d'autres ONG ou l'un de leurs mobilisateurs ? Si oui, veuillez décrire comment vous en êtes venu à vous engager avec eux et ce que vous en avez fait.

- Le personnel de Blue Ventures ou autres ONG vous a directement contacté ?
- Participation à des événements ou services de Blue Ventures ou autres ONG ?

Dans vos interactions avec Blue Ventures ou autres ONG, avez-vous pu leur dire ce que vous vouliez ou ce dont vous aviez besoin dans votre communauté ? Avez-vous déjà participé à la planification ou à la prise de décision concernant les actions entreprises ? Veuillez donner plus de détails possible.

Dans l'ensemble, que pensez-vous de l'utilité des activités de Blue Ventures et d'autres ONG pour aider votre communauté à relever les défis auxquels vous avez été confronté ?

- Y a-t-il des aspects positifs dans la façon dont Blue Ventures et d'autres ONG soutiennent la réponse aux crises dans cette communauté ? (par exemple, ont-ils écouté ce que la communauté voulait ? Les ont-ils impliqués dans la planification/prise de décision ?)
- Que pourraient faire ces ONG mieux ou différemment (y compris la façon dont elles interagissent avec les communautés et comment elles les incluent dans la prise de décision et l'élaboration de plans pour se préparer à répondre à toute crise).

Que peut-on faire pour rendre les actions durables pour la réponse aux crises climatiques/ environnementales dans cette région ?

Y a-t-il autre chose que vous aimeriez dire sur la réponse aux crises sanitaires ou climatiques auxquelles vous êtes confrontés dans cette communauté ?

Merci pour votre temps.

<u>Topic Guide KII, English version</u>

Study title: Community Engagement, Resilience, and Partnership for Health Responses in Cyclone-Affected Settings: Evidence from Madagascar

Principal Investigator: Dr. Mateus Kambale Sahani
Supervisors: Dr Neha Singh, Prof Susannah Mayhew, and Prof Janet Seeley

Topic guides KII (for stakeholders and local authorities)

<u>I. Identification</u>

ID number: ………………… **Age:** …………. (years) **Sex:** M/ F/ Others (specify):………………………..

Level of education: reached primary/ reached secondary school/ reached the university

Residency (Commune, Village):

1. Commune Ambalahonko (village: Ambiky) 2. Commune Ankatafa (village: Ankatafabe) 3. Commune Bemanevika Haute Sambirano (village: Ambodifinesy) 4. Commune Maherivaratra (village: Ampampamena) 5. Commune Ambohimena (village: Ampahakabe) 6. Others (specify): ………………..

Social status: 1. Government official 2. NGO Representative 3. Religious leader 6. Health professional 7. Others (specify): ……………………………………………………………….

II. Questions

Have you experienced (or any member of your staff) any crisis that have affected health and livelihoods such as cyclone, epidemic (apart of COVID-19), drought in this community?

- If yes, how did it affect your staff and your community members? (e.g. food/water shortages/life threatening?)
- did you do any intervention to help community members coping with this situation?
- Were any actions – by you or others – put in place to solve the issue? What were these actions and what was their purpose?
- Were these actions successful, in your view?
- If yes, what made them successful?

Have you ever collaborated with the community members to respond to health/environment crisis (epidemic, drought, flooding, cyclone, etc.) in this community?

- If yes, what were you responding to (cyclone, flood, food, or water shortage etc.)?
- How do you select which people to work with from the community?
- How did you proceed to involve community members?
  - In what circumstances (context)? What was their role?
  - Are you involving them in the planning, or would you like to involve them, in planning the response interventions?
  - Do you recruit some of the community members to implement your [NGO/Government] activities to respond to a health/climate related emergency? Who do you choose and why?
  - Have you ever invited community members for community dialogue for a crisis response?
  - Have you ever trained members of the community that you serve in how to respond to health/climate related crisis?
- Were community contributions helpful to your response mechanisms? (why/how?)
- Did you get feedback from them that informed your response mechanisms?
- Do you think they trust you?
  - If yes, what did you do to build that trust? Please explain more.
- Do you think health has improved because of the collaboration with community members?
  - If yes, how (please explain more)?

How willing do you find community members to take part in activities responding to health/environment crisis (epidemic, drought, flooding, cyclone, etc.)?

- Do you commonly work in collaboration with them?

- What do you think motivates them to be engaged in (and/or collaborate with you) on crisis response?
- Were they responsive or hesitant to contribute?
- Do they wait to be invited to participate or can they initiate the response by themselves at the community level without being invited?
- If you ever invited them, how do they respond to your invitation? What makes them responsive or hesitant?
- If they ever asked you for help, how did you respond to their invitation?

Do you get communication from official/meteorological authorities about cyclones hitting for your preparedness and to help you plan your support to the community? If yes, is it done well in advance and how do you prepare yourself and the community that you are supporting? Does it help you to plan your activities? Does it contribute to avoid loss of lives in the community?

Do you get communication from official/meteorological authorities about projection of famine crisis for your preparedness and to help you plan your support to the community? If yes, is it done well in advance and how do you prepare yourself and the community that you are supporting? Does it help you to plan your activities? Does it contribute to avoid loss of lives in the community?

Do you think any of the crises has impacted the community members economically?

- What happened?
- What did you do to help them rebuild their economy?
- Did they get support from you or any other donors?
  - If other donors, who are they?

Are you aware of the existing guidelines for the construction of wooden houses allowing resistance to cyclones or storm? If yes, how do you use them to interact with the community members to prepare them to cyclone effect? Are there people or NGO who speak to the communities to sensitise them about these guidelines? Do you think these guidelines are helpful for the community? How can they inform your interventions for the community?

How do you find the fishing areas closure or protected areas policies for the community? Do they help you in developing your programmes and support the community? How are they implemented? Are the communities involved in the implementation? Are the protected areas or the closure dates determined jointly with the communities? Do you think the policies could be implemented differently?

Is there a group of people in your community ready any time to respond to a health/climate related crisis?

- If yes, who are they, how were they established and how do they work?
- Do they have permanent activities relating to preparing for and responding to crises?
- What is being done to maintain them active and useful?
- Are they in contact with (or do they receive any support from) donors and/or government?
- If they are not there, are they needed? Why?

In your interactions with the community members, were they able to tell you what they thought should be done (what is needed for them)? Do you involve them in the planning or decision making about actions being undertaken? Please give as much detail as possible.

Overall, what is your view of how helpful are your activities or those of other NGO or government in the management of cyclones effects, to support community members to respond to the challenges you have faced during cyclones or in the immediate post-cyclone period? What do you plan for them for a long-term preparedness?

- Are there any positive aspects of how Blue Ventures or other NGOs support crisis-response in this community? (e.g. did they listen to what community wanted? Did they involve them in planning/decision making? What aspects of the interactions did your participants value most?)
- What could you do better/differently (including how you interact with communities and the extent to which you include them in decision making and developing plans for preparing to respond to any crises)

Is there anything else you would like to add on how you – or people in your communities – respond to the health crises you face?

Thank you for your time.

<u>Topic Guide KII, French version</u>

Titre de l'étude : Engagement communautaire, résilience et partenariat pour les réponses sanitaires dans les contextes touchés par les crises de cyclones : études de cas de Madagascar

Chercheur principal : Dr Mateus Kambale Sahani
Superviseurs : Dr Neha Singh, Prof Susannah Mayhew, and Prof Janet Seeley

Guides thématiques KII (pour les parties prenantes et les autorités locales)

<u>I. Identification</u>

Numéro d'identification : .... **Âge :** .... (années) **Sexe :** H/ F/ Autres (préciser) :............

Niveau d'éducation : a atteint l'école primaire / a atteint l'école secondaire / a atteint l'université

Résidence (Commune, Village) :

1. Commune Ambalahonko (village: Ambiky) 2. Commune Ankatafa (village: Ankatafabe) 3. Commune Bemanevika Haute Sambirano (village: Ambodifinesy) 4. Commune Maherivaratra (village: Ampampamena) 5. Commune Ambohimena (village: Ampahakabe) 6. Others (specify): ………………

Statut social : 1. Fonctionnaire du gouvernement 2. Représentant des ONG 3. Chef religieux
6. Professionnel de la santé 7. Autres (préciser) : ....

II. Questions

Avez-vous vécu (ou un membre de votre personnel) une crise qui a affecté la santé et les moyens de subsistance comme un cyclone, une épidémie (en dehors de COVID-19), une sécheresse dans cette communauté ?

- Si oui, comment cela a-t-il affecté votre personnel et les membres de votre communauté ? (par exemple, pénuries de nourriture/d'eau/danger de mort ?)
- Avez-vous fait une intervention pour aider les membres de la communauté à faire face à cette situation ?
- Des actions – de votre part ou de celles d'autres personnes – ont-elles été mises en place pour résoudre le problème ? Quelles étaient ces actions et quel était leur but ?
- Ces actions ont-elles été couronnées de succès, selon vous ?
- Si oui, qu'est-ce qui a fait leur succès ?

Avez-vous déjà collaboré avec les membres de la communauté pour répondre à une crise sanitaire/environnementale (épidémie, sécheresse, inondation, cyclone, etc.) dans cette communauté ?

- Si oui, à quoi avez-vous réagi (cyclone, inondation, pénurie de nourriture ou d'eau, etc.) ?
- Comment sélectionnez-vous les personnes avec lesquelles travailler dans la communauté ?
- Comment avez-vous procédé pour impliquer les membres de la communauté ?
  - Dans quelles circonstances (contexte) ? Quel était leur rôle ?
  - Les impliquez-vous dans la planification, ou souhaitez-vous les impliquer, dans la planification des interventions d'intervention ?
  - Recrutez-vous certains membres de la communauté pour mettre en œuvre vos activités [ONG/gouvernement] afin de répondre à une urgence liée à la santé ou au climat ? Qui choisissez-vous et pourquoi ?
  - Avez-vous déjà invité des membres de la communauté à dialoguer pour une réponse à une crise ?
  - Avez-vous déjà formé les membres de la communauté que vous servez à répondre aux crises liées à la santé et au climat ?
- Les contributions de la communauté ont-elles été utiles à vos mécanismes d'intervention ? (pourquoi/comment ?)
- Avez-vous reçu des commentaires de leur part qui ont éclairé vos mécanismes de réponse ?
- Pensez-vous qu'ils vous font confiance ?
  - Si oui, qu'avez-vous fait pour établir cette confiance ? Expliquez-nous davantage.
- Pensez-vous que la santé s'est améliorée grâce à la collaboration avec les membres de la communauté ?
  - Si oui, comment (veuillez expliquer davantage) ?

Dans quelle mesure trouvez-vous des membres de la communauté prêts à participer à des activités en réponse à une crise sanitaire/environnementale (épidémie, sécheresse, inondation, cyclone, etc.) ?

- Travaillez-vous généralement en collaboration avec eux ?
- Selon vous, qu'est-ce qui les motive à s'engager (et/ou à collaborer avec vous) dans la réponse aux crises ?
- Étaient-ils réceptifs ou hésitaient-ils à contribuer ?
- Attendent-ils d'être invités à participer ou peuvent-ils initier la réponse par eux-mêmes au niveau communautaire sans y être invités ?
- Si vous les avez déjà invités, comment répondent-ils à votre invitation ? Qu'est-ce qui les rend réactifs ou hésitants ?

- S'ils vous ont demandé de l'aide, comment avez-vous répondu à leur invitation ?

Recevez-vous des communications des autorités officielles/météorologiques sur les cyclones qui frappent pour votre préparation et pour vous aider à planifier votre soutien à la communauté ? Si oui, est-ce fait bien à l'avance et comment vous préparez-vous et préparez-vous la communauté que vous soutenez ? Cela vous aide-t-il à planifier vos activités ? Contribue-t-elle à éviter les pertes de vies dans la communauté ?

Recevez-vous des communications des autorités officielles/météorologiques sur la projection de la crise de famine pour votre préparation et pour vous aider à planifier votre soutien à la communauté ? Si oui, est-ce fait bien à l'avance et comment vous préparez-vous et préparez-vous la communauté que vous soutenez ? Cela vous aide-t-il à planifier vos activités ? Contribue-t-elle à éviter les pertes de vies dans la communauté ?

Pensez-vous que l'une des crises a eu un impact économique sur les membres de la communauté ?
- Que s’est-il passé?
- Qu'avez-vous fait pour les aider à reconstruire leur économie ?
- Ont-ils reçu votre soutien ou celui d'autres donateurs ?
  - Si d'autres donateurs, qui sont-ils ?

Connaissez-vous les directives existantes pour la construction de maisons en bois permettant de résister aux cyclones ou aux tempêtes ? Si oui, comment les utilisez-vous pour interagir avec les membres de la communauté afin de les préparer aux effets des cyclones ? Y a-t-il des personnes ou des ONG qui parlent aux communautés pour les sensibiliser à ces directives ? Pensez-vous que ces directives sont utiles pour la communauté ? Comment peuvent-ils éclairer vos interventions pour la communauté ?

Comment trouvez-vous les politiques de fermeture des zones de pêche ou des zones protégées pour la communauté ? Vous aident-ils à développer vos programmes et soutiennent-ils la communauté ? Comment sont-ils mis en œuvre ? Les communautés sont-elles impliquées dans la mise en œuvre ? Les aires protégées ou les dates de fermeture sont-elles déterminées conjointement avec les communautés ? Pensez-vous que ces politiques pourraient être mises en œuvre différemment ?

Y a-t-il un groupe de personnes, dans votre communauté, prêt à tout moment à répondre à une crise liée à la santé ou au climat ?
- Si oui, qui sont-ils, comment ont-ils été créés et comment fonctionnent-ils ?
- Ont-ils des activités permanentes liées à la préparation et à la réponse aux crises ?
- Que fait-on pour les maintenir actifs et utiles ?
- Sont-ils en contact avec les donateurs et/ou le gouvernement (ou reçoivent-ils un soutien de leur part) ?
- S'ils ne sont pas là, sont-ils nécessaires ? Pourquoi?

Dans vos interactions avec les membres de la communauté, ont-ils pu vous dire ce qu'ils pensaient qu'il fallait faire (ce qui est nécessaire pour eux) ? Les impliquez-vous dans la planification ou la prise de décision sur les actions entreprises ? Veuillez donner autant de détails que possible.

Dans l'ensemble, quelle est votre opinion sur l'utilité de vos activités ou de celles d'autres ONG ou gouvernements dans la gestion des effets des cyclones, pour aider les membres de la communauté à répondre aux défis auxquels vous avez été confrontés pendant les cyclones ou dans la période immédiatement après le cyclone ? Que prévoyez-vous pour eux pour une préparation à long terme ?

- Y a-t-il des aspects positifs dans la façon dont Blue Ventures ou d'autres ONG soutiennent la réponse aux crises dans cette communauté ? (par exemple, ont-ils écouté ce que la communauté voulait ? Les ont-ils impliqués dans la planification et la prise de décision ? Quels aspects des interactions vos participants ont-ils le plus appréciés ?)
- Que pourriez-vous faire mieux ou différemment (y compris la façon dont vous interagissez avec les communautés et la mesure dans laquelle vous les incluez dans la prise de décision et l'élaboration de plans pour vous préparer à répondre à toute crise)

Y a-t-il autre chose que vous aimeriez ajouter sur la façon dont vous – ou les gens de vos communautés – réagissez aux crises sanitaires auxquelles vous êtes confrontés ?

Merci pour votre temps.
